# NbVQ1 physically turns off the NbWRKY45-NbCAT2 module to promote reactive oxygen species burst and disease resistance in plants under high-potassium regime

**DOI:** 10.1093/plphys/kiag469

**Published:** 2026-07-01

**Authors:** Youwei Du, Shuanghong Wang, Guangli Liu, Jinchao Zhou, Zhonghong Feng, Zheyan Qiao, Shuang Zhang, Rong Zhang, Mark L Gleason, Qiang Yao, Hongchen Jia, Guangyu Sun

**Affiliations:** College of Plant Protection and State Key Laboratory of Crop Stress Resistance and High-Efficiency Production, Northwest A&F University, Yangling, Shaanxi 712100, China; College of Plant Protection and State Key Laboratory of Crop Stress Resistance and High-Efficiency Production, Northwest A&F University, Yangling, Shaanxi 712100, China; College of Plant Protection and State Key Laboratory of Crop Stress Resistance and High-Efficiency Production, Northwest A&F University, Yangling, Shaanxi 712100, China; College of Plant Protection and State Key Laboratory of Crop Stress Resistance and High-Efficiency Production, Northwest A&F University, Yangling, Shaanxi 712100, China; College of Plant Protection and State Key Laboratory of Crop Stress Resistance and High-Efficiency Production, Northwest A&F University, Yangling, Shaanxi 712100, China; College of Plant Protection and State Key Laboratory of Crop Stress Resistance and High-Efficiency Production, Northwest A&F University, Yangling, Shaanxi 712100, China; College of Plant Protection and State Key Laboratory of Crop Stress Resistance and High-Efficiency Production, Northwest A&F University, Yangling, Shaanxi 712100, China; College of Plant Protection and State Key Laboratory of Crop Stress Resistance and High-Efficiency Production, Northwest A&F University, Yangling, Shaanxi 712100, China; Department of Plant Pathology, Entomology, and Microbiology, Iowa State University, Ames, IA 50011, United States; Qinghai Academy of Agriculture and Forestry Science, Qinghai University, Xining 810016, China; College of Plant Protection and State Key Laboratory of Crop Stress Resistance and High-Efficiency Production, Northwest A&F University, Yangling, Shaanxi 712100, China; State Key Laboratory of Tropical Crop Breeding, Institute of Tropical Bioscience and Biotechnology & Sanya Research Institute, Chinese Academy of Tropical Agricultural Sciences, Sanya 572024, China; College of Plant Protection and State Key Laboratory of Crop Stress Resistance and High-Efficiency Production, Northwest A&F University, Yangling, Shaanxi 712100, China

## Abstract

Potassium (K) nutrition is a critical determinant of plant disease resistance, but the molecular mechanisms linking K status to defense activation remain unclear. Presently, we systematically investigated the detailed mechanisms by NbVQ1-NbWRKY45-NbCAT2 module in regulating plant immunity under different K statuses. Typically, elevating K content to high (sufficient level) status enhanced pathogen-induced immune responses, particularly reactive oxygen species burst, thereby conferring broad-spectrum resistance in *Nicotiana benthamiana*. Central to this process was the NbVQ1-NbWRKY45-NbCAT2 module, which functioned as a switch for pathogen-triggered reactive oxygen species burst. In low-K plants, *NbWRKY45* was upregulated through a self-amplifying loop, thereby inducing *NbCAT2* expression to scavenge reactive oxygen species and ultimately compromising plant disease resistance. In contrast, adding K promoted *NbVQ1* expression, which proved essential for the K-enhanced plant resistance. NbVQ1 interacted with the WRKYGQK domain of NbWRKY45 through its VQ motif, thereby disrupting the binding affinity of NbWRKY45 to *NbCAT2* promoter and causing *NbCAT2* downregulation. This transcriptional suppression of *NbCAT2* resulted in stronger reactive oxygen species bursts and improved the resistance of *N. benthamiana* to *Botrytis cinerea* and *Phytophthora parasitica* under high-K status. Notably, K^+^ could promote the interaction between NbVQ1 and NbWRKY45, directly explicating nutrient-associated immune potentiation mechanism. Moreover, this K-dependent resistance mechanism mediated by VQ1-WRKY45-CAT2 module was conserved in *Arabidopsis thaliana*, while suppression of NbWRKY45 also increased plant drought tolerance. Overall, this study established the VQ-WRKY-CAT module as a molecular switch for K-promoted reactive oxygen species immunity and provided mechanistic evidence for coupling K nutrition with transcriptional regulation of plant immunity.

## Teaser

NbVQ1 suppresses the NbWRKY45-NbCAT2 module to promote reactive oxygen species burst and immune responses for enhancing disease resistance of plants with high-potassium status.

## Introduction

Potassium (K) is an indispensable macronutrient for plant growth and development due to its essential roles in enzyme activation, cellular homeostasis, and metabolite biosynthesis (Wang and Wu 2010). Sufficient K supplementation not only secures crop yield but also reduces the incidence of plant diseases (Amtmann et al. 2008). For instance, K fertilization sufficient to maintain nutrient levels in apple (*Malus domestica*) can effectively control the Apple Valsa canker (Peng et al. 2016; Du et al. 2023b). The addition of K enhances basal resistance in plants by upregulating resistance-related genes and alleviating lipid peroxidation (Shi et al. 2018). Additionally, it has been shown that pathogen elicitors trigger rapid K^+^ release from plant cells, accompanied by Ca^2+^ influx and H^+^ efflux, and this K^+^ efflux is closely linked to the generation of reactive oxygen species (ROS), which further activated plant defensive responses (Demidchik et al. 2014; Shabala et al. 2014). Meanwhile, changes in K content also affect the signal transduction regulating plant defense response to a range of abiotic and biotic stresses (Shabala et al. 2014; Shabala 2017). Despite these advances, the regulatory mechanisms underlying resistance responses in plants under high-K (HK) conditions remain unclear.

Plants are constantly exposed to various biotic challenges, particularly by pathogenic microbes (Dodds and Rathjen 2010). Confrontation with pathogens has driven the evolution of sophisticated defense systems, including R gene–mediated resistance and basal resistance (Ngou et al. 2022a). To ensure resistance effectiveness, plants employ precise signaling pathways such as mitogen-activated protein kinases (MAPKs) cascades, transcriptional modules, and ROS burst signaling (Ngou et al. 2022b; Jones et al. 2024). As critical defensive mediators, ROS are mainly produced by apoplastic NADPH oxidases Respiratory burst oxidase homologs (RBOHs) and act as immune signals with antifungal activities (Wong et al. 2007; Mittler et al. 2022). Because excessive ROS accumulation is toxic to host cells, plants employ enzymatic scavengers such as catalase (CAT) to maintain ROS homeostasis and prevent damage (Apel and Hirt 2004). In addition, ROS contributes to the resistance to abiotic stresses, such as osmotic and salt challenges (Locascio et al. 2019; Zhu et al. 2024). Notably, ROS production is closely associated with changes in plant K levels, and K supplementation promotes stomatal closure by inducing ROS generation, thereby conferring plant adaptation to environmental challenges (Khokon et al. 2011; Osakabe et al. 2013; Locascio et al. 2019). Moreover, pathogen-elicited K^+^ efflux has been observed in multiple plant–pathogen interactions, and blocking K^+^ channels can prevent ROS production and downstream defense responses in plants (Demidchik et al. 2010). These findings suggest that ROS production may serve as a critical link between plant disease resistance and K nutritional status.

Plants employ systematic regulatory pathways, including the VQ-WRKY transcriptional module, to mediate the expression of genes involved in ROS production and scavenging (Niu et al. 2020; Liu et al. 2021). WRKY transcription factors constitute the largest family in plants, defined by a highly conserved WRKYGQK motif that binds to W-boxes within gene promoters (Cheng et al. 2012). WRKYs form complex regulatory networks orchestrating the expression of genes involved in plant development and resistance responses (Jiang et al. 2017; Du et al. 2023a; Yang et al. 2025). For example, TaWRKY19 transcriptionally suppresses *TaNOX10* expression, thereby reducing ROS production and wheat resistance to stripe rust (Wang et al. 2022). Additionally, VQ proteins containing the conserved FxxxVQxLTG motif also emerged as important negative or positive regulators of plant defense against diverse pathogens and have been characterized as key cofactors that modulate the transcriptional activity of WRKYs through direct physical interactions (Chen et al. 2018; Hao et al. 2022). For instance, VQ23/SIB1 activates resistance to *Pseudomonas syringae*, whereas VQ20 negatively regulates resistance to both biotrophic and necrotrophic pathogens (Xie et al. 2010). Moreover, VQ proteins are involved in responses to oxidative stress and hypoxia, as many VQ genes are induced by low-oxygen conditions (Cheng et al. 2012). Notably, ROS and nitric oxide (NO) have been linked to VQ-WRKY module regulation, with specific VQ-WRKY complexes controlling ROS production under various stress conditions (Van Breusegem and Dat 2006). Previous studies have identified numerous genes encoding VQs and WRKYs as being highly expressed in HK *Nicotiana benthamiana* (Du et al. 2024b), suggesting the involvement of the VQ-WRKY module in regulating plant basal resistance under HK conditions.

In this study, the regulatory mechanisms of basal resistance in *N. benthamiana* under different K conditions were systematically investigated. Notably, NbVQ1-NbWRKY45-NbCAT2 transcriptional module that functioned as molecular switch for ROS homeostasis and basal defense was identified as a conserved mechanistic link between K nutrient status and plant resistance. It was demonstrated that maintaining sufficient K levels ensured the full activation of ROS burst and resistance responses through the specific deployment of NbVQ1, which disrupted the NbWRKY45-NbCAT2 axis otherwise responsible for ROS scavenging in plants. This direct coupling of nutrient availability with a transcriptional complex controlling ROS burst highlights the importance of the “plant nutrient immunity” concept in crop disease management. Therefore, these findings provide a theoretical basis for optimizing management of nutrient elements, especially K nutrient, to enhance broad-spectrum crop resilience against pathogenic threats in agriculture system.

## Results

### Potassium supplementation promotes disease resistance and *NbVQs* expression in *N. benthamiana*

**Figure 1:**
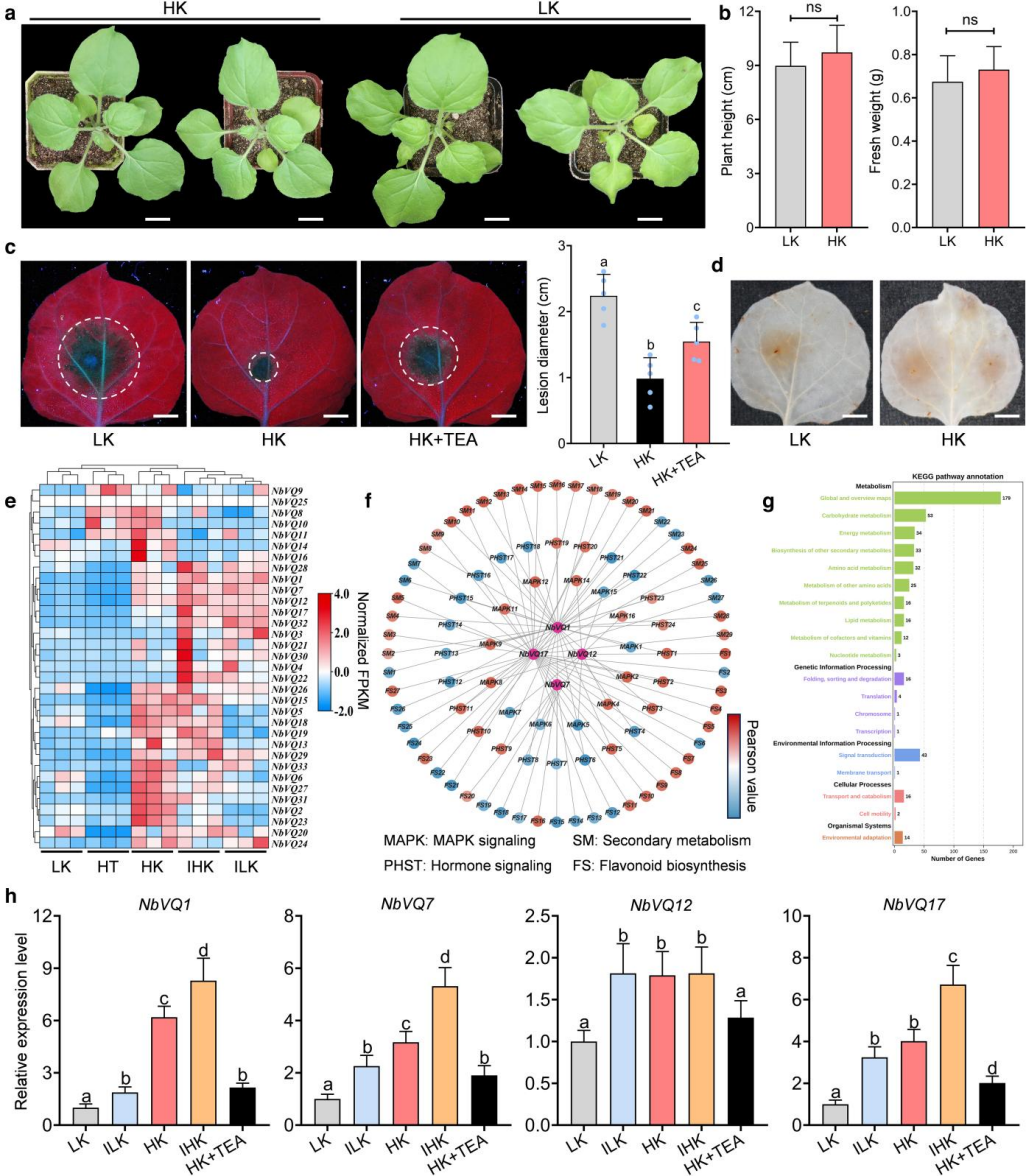
Potassium (K) supplementation promotes *NbVQs* expression and confers resistance of *N. benthamiana* to *P. parasitica*. a) Phenotype of *N. benthamiana* plants grown under HK and LK conditions. b) Plant height and fresh weight of HK and LK *N. benthamiana* seedlings; *n* = 5. c) Enhanced resistance of *N. benthamiana* to *P. parasitica* under HK conditions; *n* = 5. d) Increased production of *P. parasitica*–induced ROS (H_2_O_2_) in *N. benthamiana* under HK conditions. e) Heat map displaying the expression profiles of *NbVQs* in infected and noninfected *N. benthamiana* under HK or LK conditions. The scale bar represents the normalized gene fragments per kilobase of transcript per million mapped reads (FPKM) value in each biological replicate. f) Coexpression regulatory network of *NbVQs* and resistance-related genes in *N. benthamiana*. The scale bar represents the Pearson value between 2 nodes. g) KEGG enrichment analysis of genes highly correlated with *NbVQ1*, *NbVQ7*, *NbVQ12*, and *NbVQ17* in the regulatory network. h) RT-qPCR validation of expression levels of *NbVQ1*, *NbVQ7*, *NbVQ12*, and *NbVQ17* in infected and noninfected *N. benthamiana* under HK and LK conditions; *n* = 3. Data are presented as the mean ± Sd. Different letters in c and h) indicate significant differences based on *P* < 0.05 (1-way ANOVA with Tukey's post hoc test); “ns” in b) represents no significance (Student's *t*-test).

Previous studies have demonstrated that increasing K content enhances disease resistance in both apple and *N. benthamiana* (Du et al. 2023b, 2024b). To further investigate the K-associated mechanisms underlying plant resistance, *N. benthamiana* was cultivated under HK and low-K (LK) conditions to obtain HK (sufficient K level; 42.8 ± 1.7 g/kg) and LK (insufficient K level; 22.0 ± 2.6 g/kg) seedlings according to its physiological standard (Fig. S1a; Table S1; Chouteau and Fauconnier 1998). No significant differences in physiological traits, including plant height, fresh weight, and dry weight, were observed between HK and LK plants (Figs. 1a and b and S1a and b). Inoculation with *Phytophthora parasitica* and *Botrytis cinerea* produced distinct lesion symptoms in both groups. At the 2-d postinoculation (dpi), HK plants exhibited markedly higher resistance than LK plants (Fig. 1c). In contrast, tetraethylammonium (TEA) treatment reduced both K content and resistance in *N. benthamiana* even under HK conditions, confirming the role of K in enhancing plant disease resistance (Fig. 1c; Table S1). Moreover, the effects of adding K nutrient in promoting plant disease resistance exerted a dose-dependency (Fig. S2a; Table S1). Notably, ROS production and callose deposition in HK plants were significantly higher than those in LK plants following *P. parasitica* infection (Figs. 1d and S1c), highlighting the effect of K supplementation on the activation of resistance events. Reverse transcription quantitative PCR (RT-qPCR) and transient expression assays demonstrated that among the tested *NbRBOHs*, only *NbRBOHD* and *NbRBOHF* were upregulated in HK plants and could be induced by *P. parasitica* infection (Fig. S1d). Further transient expression assays showed that *NbRBOHD* overexpression played a more prominent role than *NbRBOHF* in conferring enhanced ROS burst and resistance in *N. benthamiana* under HK conditions (Fig. S1e to g). These results suggested that the NbRBOHD-produced ROS burst was important for defense response of *N. benthamiana* under HK conditions.

Subsequently, transcriptome comparisons between HK and LK plants revealed that K supplementation combined with *P. parasitica* infection upregulated multiple *NbVQs*, an effect suppressed by TEA treatment even under HK conditions (Fig. 1e). Coexpression network identified *NbVQ1*, *NbVQ7*, *NbVQ12*, and *NbVQ17* as hub regulators of K-associated resistance responses, especially phenylpropanoid biosynthesis and MAPK signaling (Figs. 1f and g and S1h; Table S2). Further RT-qPCR validation confirmed that *NbVQ1*, *NbVQ7*, and *NbVQ17* were strongly induced by both K supplementation and pathogen infection, with *NbVQ1* demonstrating the most pronounced upregulation in HK plants (Fig. 1h). Moreover, TEA-caused decreases in K content significantly reduced the upregulation magnitude of *NbVQ1*, *NbVQ7*, and *NbVQ17* in HK plants, supporting the effects of adding K nutrient in activating these *NbVQs* (Fig. 1h). Collectively, these results suggest that *NbVQ1*, *NbVQ7*, and *NbVQ17* contribute to HK-associated basal resistance in *N. benthamiana*.

### NbVQ1 is essential for the disease resistance of HK *N*. *benthamiana* to pathogen

**Figure 2:**
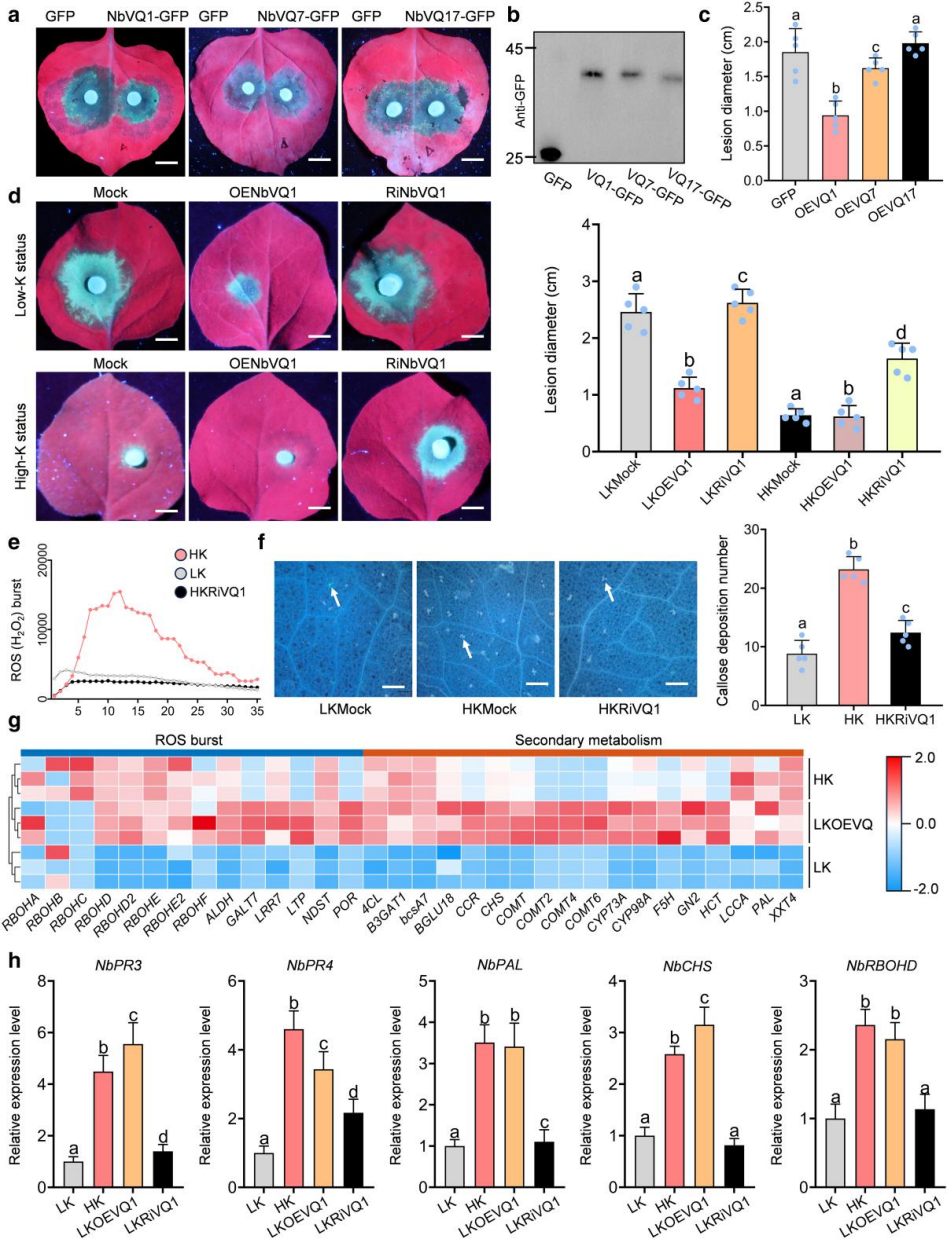
NbVQ1 mediates enhanced disease resistance of HK *N*. *benthamiana* to *P. parasitica*. a and c) Effects of *NbVQ1*, *NbVQ7*, and *NbVQ17* overexpression on *N. benthamiana* resistance to *P. parasitica*. The lesion diameter is determined at 2 d post inoculation of *P. parasitica*; GFP is used as control; *n* = 5. b) Western blotting confirming transient overexpression of *NbVQ1*, *NbVQ7*, and *NbVQ17* in representative *N. benthamiana*. d) NbVQ1 is responsible for conferring HK-associated high resistance in *N. benthamiana*; *n* = 5. e) RNAi-mediated *NbVQ1* silencing suppresses the flg22-induced ROS (H_2_O_2_) burst in HK *N. benthamiana*; *n* = 3. f) Levels of *P. parasitica*–induced callose deposition in HK and LK *N. benthamiana* with *NbVQ1* silencing. The callose deposits are labeled using white arrow; bar = 50 *μ*m; *n* = 5. g) Heat map showing the expression profiles of genes associated with ROS burst and secondary metabolism in HK, LKOEVQ, and LK *N. benthamiana*. The scale represents the normalized gene FPKM values. h) Expression levels of resistance-related genes (*NbPAL*, *NbCHS*, *NbRBOHD*, *NbPR3*, and *NbPR4*) in LK and HK *N. benthamiana* with *NbVQ1* overexpression or silencing; *n* = 3. Data are presented as the mean ± Sd. Different letters represent significant differences at *P* < 0.05 based on 1-way ANOVA followed by post hoc Tukey test.

Here, we found that *NbVQ1* upregulation exerted a dose-dependent character on the increases of K content in *N. benthamiana*, and transient overexpression of *NbVQ1* that predominantly localized in the nucleus produced the strongest enhancement of resistance (Figs. 2a to c, S2b, and S3). Meanwhile, its homologs (NbVQ2 and NbVQ3) did not exhibit expression changes in response to *P. parasitica* infection in *N. benthamiana* under different K statuses (Fig. S4a to c). To elucidate the functional contribution of NbVQ1 to K-dependent basal resistance, transgenic *N. benthamiana* lines were established under both HK and LK conditions, in which *NbVQ1* was either stably silenced or overexpressed (Figs. 2d and S5a to d). In LK plants, *NbVQ1* overexpression (LKOEVQ1) markedly enhanced resistance to both *P. parasitica* and *B*. *cinerea*, producing significantly smaller lesions than in LKMock (Figs. 2d and S5a to e). Conversely, silencing *NbVQ1* through RNAi markedly reduced the resistance of HK *N. benthamiana* to both *P. parasitica* and *B*. *cinerea* (HKRiVQ1), underscoring its essential role in mediating the K-associated broad-spectrum resistance (Figs. 2d and S5a to e). In HKRiVQ1 plants, the pathogen challenge resulted in a diminished ROS burst and impaired callose deposition, indicating that the suppression of *NbVQ1* disrupted basal defense even under HK conditions (Fig. 2e and f).

Subsequently, transcriptome profiles revealed that *NbVQ1* overexpression reshaped the transcription pattern of LK *N. benthamiana* (LKOEVQ; Fig. S6a and b). And, the NbVQ1-upregulated genes functioned in numerous basal resistance pathways, including phenylpropanoid and flavonoid biosynthesis, MAPK signaling, and plant–pathogen interaction (Fig. S6c; Tables S3 and S4). Moreover, genes associated with ROS burst, phenylpropanoid biosynthesis, and cell wall organization were highly expressed in both HK and LKOEVQ plants, including *NbPAL*, *NbCHS*, *NbLccA*, *NbCOMT*, *NbHCT*, *NbRBOHD*, *NbRBOHF*, and *NbRBOHE* (Figs. 2g and S6d). Furthermore, RT-qPCR analysis confirmed that *NbVQ1* overexpression in LK plants enhanced the transcription of key resistance genes, including *NbPAL*, *NbCHS*, *NbRBOHD*, and *NbPRs*, whereas its silencing in HK plants led to their suppression (Fig. 2h). These results indicated that *NbVQ1* overexpression activated HK-associated resistance responses represented by ROS burst and defensive metabolism in *N. benthamiana*.

Previous studies have reported that secondary metabolites, especially phenylpropanoids, act as critical defensive metabolites in plant pathogen resistance (Xia et al. 2021). Given the involvement of secondary metabolism in NbVQ1- and K-associated resistance, the metabolic profiles of LK, LKOEVQ, and HK plants were established (Table S5). Both the principal component analysis (PCA) and unsupervised correlation analyses revealed distinct metabolic signatures across these groups, while numerous defensive metabolisms producing flavonoid, phenylpropanoid, glucosinolate, and terpenoid were more active in LKOEVQ (Fig. S6e to g; Table S6). Consistently, the majority of related metabolites accumulated at higher levels in HK and LKOEVQ plants than in LK plants (Fig. S6h). Collectively, these findings indicate that *NbVQ1* drives K-associated activation of basal resistance events represented by ROS burst and defensive metabolisms in HK *N. benthamiana*.

### NbWRKY45 physically interacts with NbVQ1 in the nucleus and reduces resistance of *N. benthamiana* to *P. parasitica*

**Figure 3:**
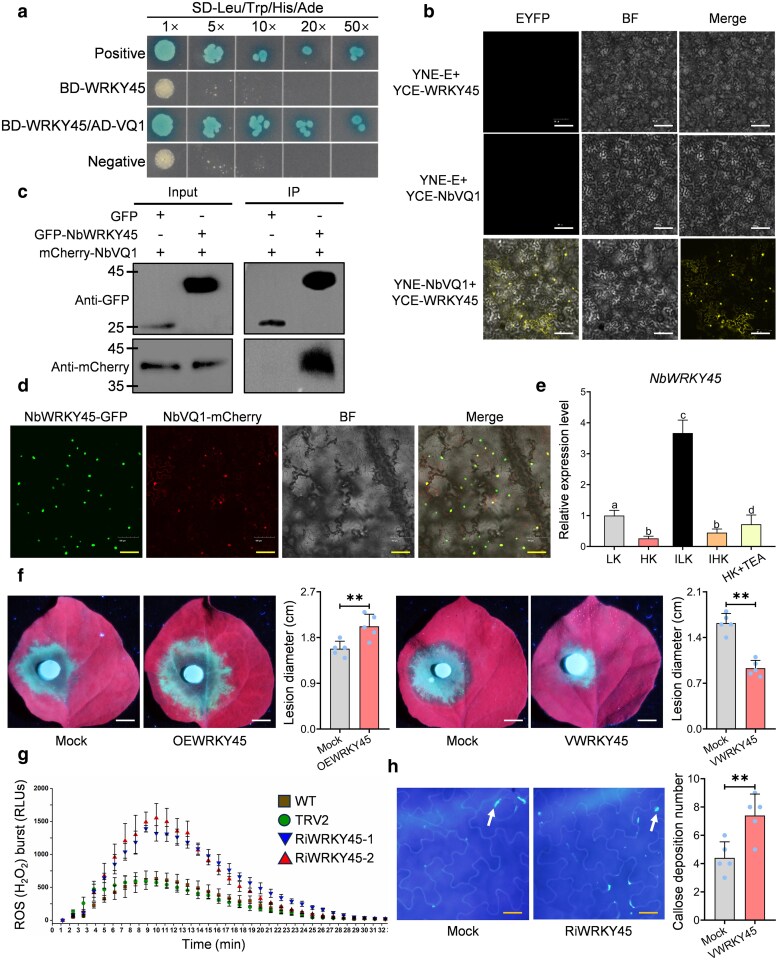
NbWRKY45 physically interacts with NbVQ1 and negatively regulates basal resistance in *N. benthamiana*. a) Y2H assay showing the interaction between NbVQ1 and NbWRKY45. The Y2HGold strain containing AD-empty or AD-NbVQ1 and BD-empty or BD-NbWRKY45 is grown on SD-Leu/Trp/His/Ade selective medium amended with 150 ng/*μ*L AbA and 10 ng/*μ*L X-α-gal. The yeast cells exhibiting beta-galactosidase activity reveal interaction between NbVQ1 and its targets. b) BiFC assay showing the interaction between NbVQ1 and NbWRKY45 in the nuclei of *N. benthamiana*; bar = 20 *μ*m. *NbVQ1* and *NbWRKY45* are fused to the C-terminal region of the YFP (YCE-NbVQ1) and the N-terminal YFP region (YNE-NbWRKY45), respectively. *Agrobacterium* strains containing YCE-NbVQ1 and YNE-NbWRKY45 are coinfiltered in *N. benthamiana*. c) Co-IP assay confirming the interaction between NbVQ1 and NbWRKY45 in vivo. Total proteins are extracted and subjected to immunoprecipitation of NbWRKY45 by GFP antibeads. The input proteins are detected using anti-GFP and anti-mCherry antibodies. d) Subcellular colocalization of NbVQ1-mCherry and NbWRKY45-GFP in the nuclei of *N. benthamiana*. Both *Agrobacterium* strains containing NbVQ1-mCherry or NbWRKY45-GFP are coinfiltrated in *N. benthamiana* for observation using laser scanning confocal microscope; bar = 20 *μ*m. e) RT-qPCR analysis of *NbWRKY45* expression in infected and noninfected *N. benthamiana* under HK and LK conditions; *n* = 3. f) NbWRKY45 negatively regulates resistance of *N. benthamiana* to *P. parasitica*. The expression of *NbWRKY45* was silenced using VIGS system; *n* = 5. g) RNAi-mediated *NbWRKY45* silencing promotes the level of flg22-triggered ROS (H_2_O_2_) burst in *N. benthamiana*; *n* = 3. h) *NbWRKY45* silencing increases flg22-triggered callose deposition in *N. benthamiana*; bar = 20 *μ*m; *n* = 5. Data are presented as the mean ± Sd. Different letters and “**” represent significant differences based on *P* < 0.05. Statistical analysis in e) is determined by 1-way ANOVA followed by post hoc Tukey test, and Student's *t*-test in f and h).

Here, IP-MS was performed to identify potential interactors of NbVQ1 in *N. benthamiana*, and multiple NbWRKYs were observed to associate with NbVQ1 (Table S16). Further yeast 2-hybrid (Y2H) assay showed that yeast cells expressing AD-NbVQ1 and BD-NbWRKY45 exhibited growth and α-galactosidase activity on -Trp/-His/-Leu/-Ade medium, indicating the physical interaction between NbVQ1 and NbWRKY45 (Fig. 3a). Bimolecular fluorescence complementation (BiFC) and colocalization assays further demonstrated the nuclear interaction of NbVQ1 with NbWRKY45 (Fig. 3b and d), while co-immunoprecipitation (Co-IP) assays confirmed their association in vivo (Fig. 3c).

To clarify the role of NbWRKY45 in the K- and NbVQ1-mediated resistance responses, its functional characteristics were examined. Sequence analysis revealed that NbWRKY45 shared homology with AtWRKY45, AtWRKY75, and OsWRKY72, with the highest conservation in the C-terminal amino acid region (Fig. S7a and b). And, *NbWRKY45* expression was activated by methyl jasmonate (MeJA) and flagelin 22 (flg22) but suppressed by salicylic acid (SA) treatment in vivo (Fig. S7c). Notably, *NbWRKY45* expression was markedly elevated in LK *N. benthamiana*, and induced to upregulate by TEA-caused decreases in K content in *N. benthamiana*, even under HK conditions (Fig. 3e). Although *P. parasitica* infection induced *NbWRKY45* expression in LK *N. benthamiana*, adding in planta K content could substantially reduce *NbWRKY45* expression in a dose-dependency manner (Figs. 3e and S2b). Importantly, *NbWRKY45* overexpression significantly compromised resistance in *N. benthamiana*, whereas its silencing by virus-induced gene silencing (VIGS) system enhanced resistance to *P. parasitica* (Figs. 3f and S8a to d). Meanwhile, RNAi-mediated silencing of *NbWRKY45* also intensified ROS burst and callose deposition upon flg22 stimulation compared with mocks (Figs. 3g and h and S8a to d), indicating that *NbWRKY45* upregulation attenuated basal resistance. Overall, these findings demonstrated that NbWRKY45 physically interacts with NbVQ1 and acts as a negative regulator of basal resistance in *N. benthamiana*.

### NbWRKY45 negatively regulates K-associated disease resistance in *N. benthamiana*

**Figure 4:**
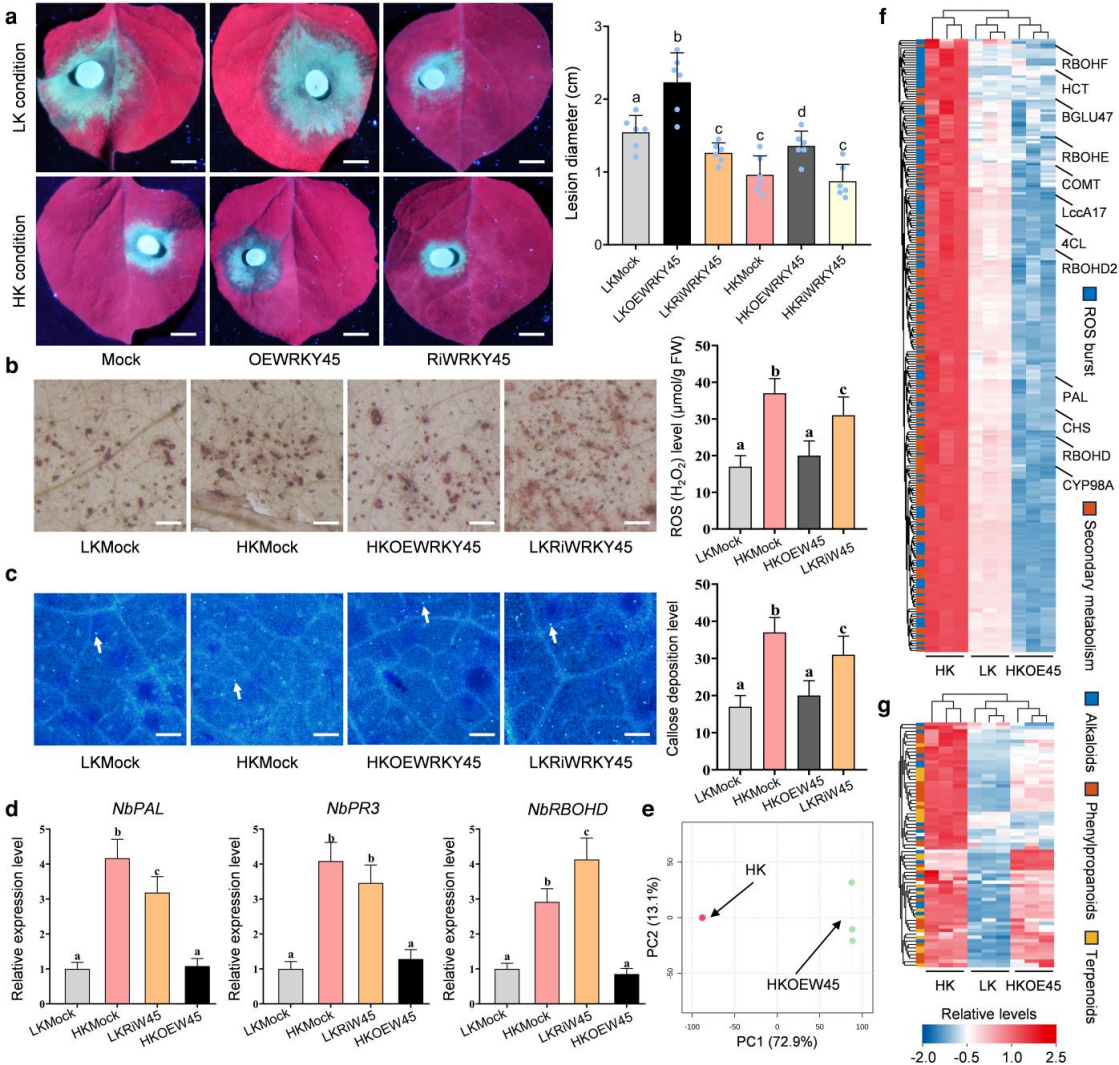
NbWRKY45 attenuates resistance in *N. benthamiana* to *P. parasitica* under LK conditions. a) *NbWRKY45* upregulation was associated with reduced resistance in *N. benthamiana* under LK conditions; *n* = 6. b) Histochemical detection of *P. parasitica*–induced ROS (H_2_O_2_) production in HK and LK *N. benthamiana* following *NbWRKY45* silencing or overexpression; bar = 100 mm; *n* = 3. c) *NbWRKY45* upregulation causes decreases in *P. parasitica*–induced callose deposition in *N. benthamiana*; bar = 100 *μ*m; *n* = 3. d) RT-qPCR analysis of resistance-related gene expression in HK and LK *N. benthamiana* after *NbWRKY45* silencing or overexpression; bar = 50 *μ*m; *n* = 3. e) PCA plots displaying distinct transcription patterns between HKOEWRKY45 and HKMock *N. benthamiana*. f) Heatmap showing the suppressive effects of *NbWRKY45* overexpression on genes involved in ROS burst and secondary metabolism in HK *N. benthamiana*. Scale bar represents the normalized FPKM values. g) Heatmap of relative abundances of defense-related metabolites in HK, LK, and HKOEWRKY45 *N. benthamiana*. Data are presented as the mean ± Sd. Different letters represent significant differences at *P* < 0.05 based on 1-way ANOVA followed by post hoc Tukey test.

Here, transgenic *N. benthamiana* lines with stable *NbWRKY45* overexpression and RNAi-mediated silencing were constructed under HK and LK conditions (HK/LKRiWRKY45 and HK/LKOEWRKY45; Figs. 4a and S8a to d). The pathogen inoculation assays revealed that the *NbWRKY45* overexpression markedly decreased the resistance of HK *N. benthamiana* to both *P. parasitica* and *B. cinerea*, and further increased the susceptibility of LK *N. benthamiana* (Figs. 4a and S8a to e). In contrast, lesion diameters in HKRiWRKY45 and LKRiWRKY45 were significantly smaller than those in HKMock and LKMock, respectively (Figs. 4a and S8a to e). The ROS burst and callose deposition in HK *N. benthamiana* were substantially suppressed by *NbWRKY45* overexpression, whereas *NbWRKY45* silencing resulted in higher levels of both responses in LK *N. benthamiana* than in LKMock (Fig. 4b and c). Moreover, *NbWRKY45* overexpression strongly inhibited the upregulation of resistance genes in HK *N. benthamiana* following *P. parasitica* infection, whereas these genes were effectively activated by *P. parasitica* in LKRiWRKY45 even under LK conditions (Fig. 4d). These results indicate that *NbWRKY45* negatively regulates K-associated disease resistance in *N. benthamiana*, with its upregulation causing increased susceptibility in LK *N. benthamiana* compared with HK plants.

Subsequently, the transcriptome profiles revealed significant differences in the transcription patterns between HKOEWRKY45 and HKMock (Figs. 4e and S9a), demonstrating the role of NbWRKY45 in reshaping the global transcriptional landscape of *N. benthamiana*. Functional enrichment analysis demonstrated that the downregulated DEGs (differentially expressed genes) from HKOEWRKY45 versus HK were categorized into series resistance pathways, especially plant-pathogen interaction, secondary metabolism and the MAPK signaling pathway (Fig. S9b to e; Tables S7 and S8). And, *NbWRKY45* overexpression markedly suppressed the expression of genes related to the ROS burst and phenylpropanoid biosynthesis in HK *N. benthamiana*, including *NbPAL*, *NbCHS*, *Nb4CL*, *NbCOMT*, *NbRBOHD*, *NbRBOHE*, and *NbRBOHF* (Fig. 4f). This supported the role of NbWRKY45 in interfering with K-associated basal resistance. Further metabolomic profiling revealed that NbWRKY45 could reprogram the K-associated metabolic composition of *N. benthamiana*, and its overexpression dramatically reduced the contents of a bunch of defensive metabolites represented by phenylpropanoids and terpenoids in HK *N. benthamiana* (Figs. 4g and S10a to e; Table S9). Specifically, *NbWRKY45* overexpression significantly reduced the levels of antimicrobial metabolites scopolin and chlorogenic acid, which were elevated by *NbVQ1* overexpression and K supplementation (Fig. S10f and g). In summary, these findings demonstrated that NbWRKY45 negatively regulates HK-associated basal resistance by suppressing defense gene expression and reducing metabolite accumulation.

### NbWRKY45 directly activates *NbCAT2* to suppress ROS burst and promote susceptibility in *N. benthamiana*

**Figure 5:**
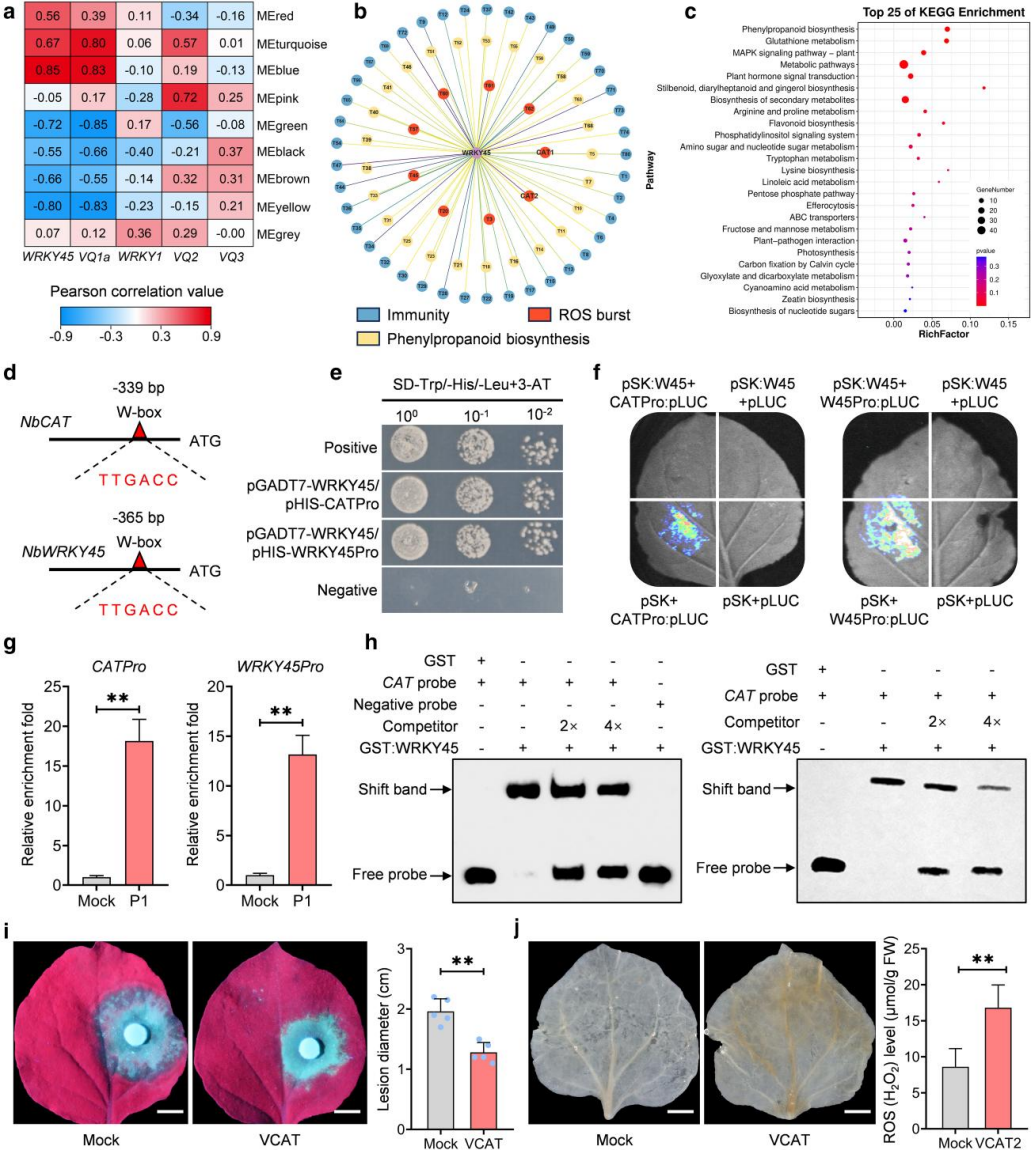
NbWRKY45 transcriptionally activates *NbCAT2* to modulate ROS accumulation. a) Construction of a regulation module for *NbVQ1* and *NbWRKY45* expression using weighted correlation network analysis (WGCNA). The column represents a trait (*NbVQ1* and *NbWRKY45* expression pattern), and the correlation value between modules and traits is shown in each cell. b) Coexpression regulation network of NbWRKY45, NbVQ1, and resistance-related genes in *N. benthamiana*. c) Scatter plot displaying pathway enrichment of genes coexpressed with *NbVQ1* and *NbWRKY45*. d) Prediction of WRKY-binding cis-elements in the promoters of *NbCAT2* and *NbWRKY45* using the PlantCARE database. e) Yeast 1-hybrid (Y1H) assay showing the interaction of NbWRKY45 with its own promoter and the *NbCAT2* promoter. Yeast cells are cotransformed with a bait vector, containing *NbCAT2*/*NbWRKY45* promoter fused to a HIS2 reporter gene, and a prey vector (pGADT7), containing *NbWRKY45*, and then grown in SD/−Leu/−Trp/-His+3-AT plates. f) Dual-LUC reporter assay revealing activation of NbWRKY45 and *NbCAT2* promoters by *NbWRKY45*. Coexpression of pSK-NbWRKY45 and NbCAT2/NbWRKY45Pro-LUC could trigger the fluorescence in *N. benthamiana*. g) Enrichment of *NbCAT2*/*NbWRKY45* promoter by NbWRKY45 in vivo. ChIP assay is performed with anti-GFP antibody using the transgenic *N. benthamiana* of OENbWRKY45. The wild type of *N. benthamiana* is used as mock. h) EMSA verifying the binding activity of NbWRKY45 to *NbCAT2*/*NbWRKY45* promoters in vitro. EMSA is performed in the presence (+) or absence (−) of NbWRKY45 and specific probe and/or unlabeled probes. The unlabeled P1 probe of *NbCAT2*/*NbWRKY45* is used as competitors. i) Transient silencing of *NbCAT2* increased resistance of *N. benthamiana* to *P. parasitica*; *n* = 5. j) *NbCAT2* silencing causes decreases *P. parasitica*–induced ROS (H_2_O_2_) accumulation in *N. benthamiana*; *n* = 3. Data are presented as the mean ± Sd. Statistical significance is represented by “**” at *P* < 0.01 based on Student's *t*-test.

To elucidate the underlying mechanisms of NbWRKY45 in mediating ROS burst, a coexpression regulatory network of *NbWRKY45* was constructed based on transcriptome profiles using the weighted gene coexpression network analysis (WGCNA; Fig. 5a and b). Genes from the MEblue module were highly correlated with *NbWRKY45* expression and were mainly involved in ROS production, immunity, phenylpropanoid biosynthesis, MAPK cascade, and plant–pathogen interaction (Fig. 5b and c). Topological network analysis identified *NbCAT2* as a hub gene within the NbWRKY45-regulated network (Fig. 5b). Further PlantCARE analysis identified a WRKY-recognized W-box (TTGACC) located in the −339 bp region of the *NbCAT2* promoter (Fig. 5d; Table S11). Yeast 1-hybrid (Y1H) and luciferase (LUC) assays subsequently demonstrated that NbWRKY45 transcriptionally activated *NbCAT2* by binding to its promoter in vivo (Fig. 5e and f). Chromatin immunoprecipitation-quantitative PCR (ChIP-qPCR) analysis further confirmed that the promoter regions of *NbCAT2* containing the W-box were significantly enriched by NbWRKY45-GFP, indicating specific binding of NbWRKY45 to the W-box in the *NbCAT2* promoter (Fig. 5g). Consistently, electrophoretic mobility shift assay (EMSA) verified that NbWRKY45 bound to the W-box region of the *NbCAT2* promoter in vitro (Fig. 5h). Functional assays further revealed that silencing of *NbCAT2* through the VIGS system enhanced pathogen-induced ROS production and resistance of *N. benthamiana* to *P. parasitica* (Figs. 5i and j and S11a to d).

Transcription factors typically possess self-activation capacity by binding to their own promoters (Zhou et al. 2020). Consistently, the prediction analysis identified a W-box in the *NbWRKY45* promoter, suggesting the self-activation potential of *NbWRKY45* in vivo (Fig. 5d; Table S12). Y1H and LUC reporter assays indicated that NbWRKY45 bound to its own promoter (Fig. 5e and f), and this interaction was further validated by ChIP-qPCR and EMSA assays both in vivo and in vitro (Fig. 5g and h). These results indicate that NbWRKY45 forms a self-activation loop to induce *NbCAT2* expression, thereby reducing ROS burst and compromising resistance in *N. benthamiana*.

### NbCAT2 scavenges ROS to reduce resistance in *N. benthamiana* under LK conditions

**Figure 6:**
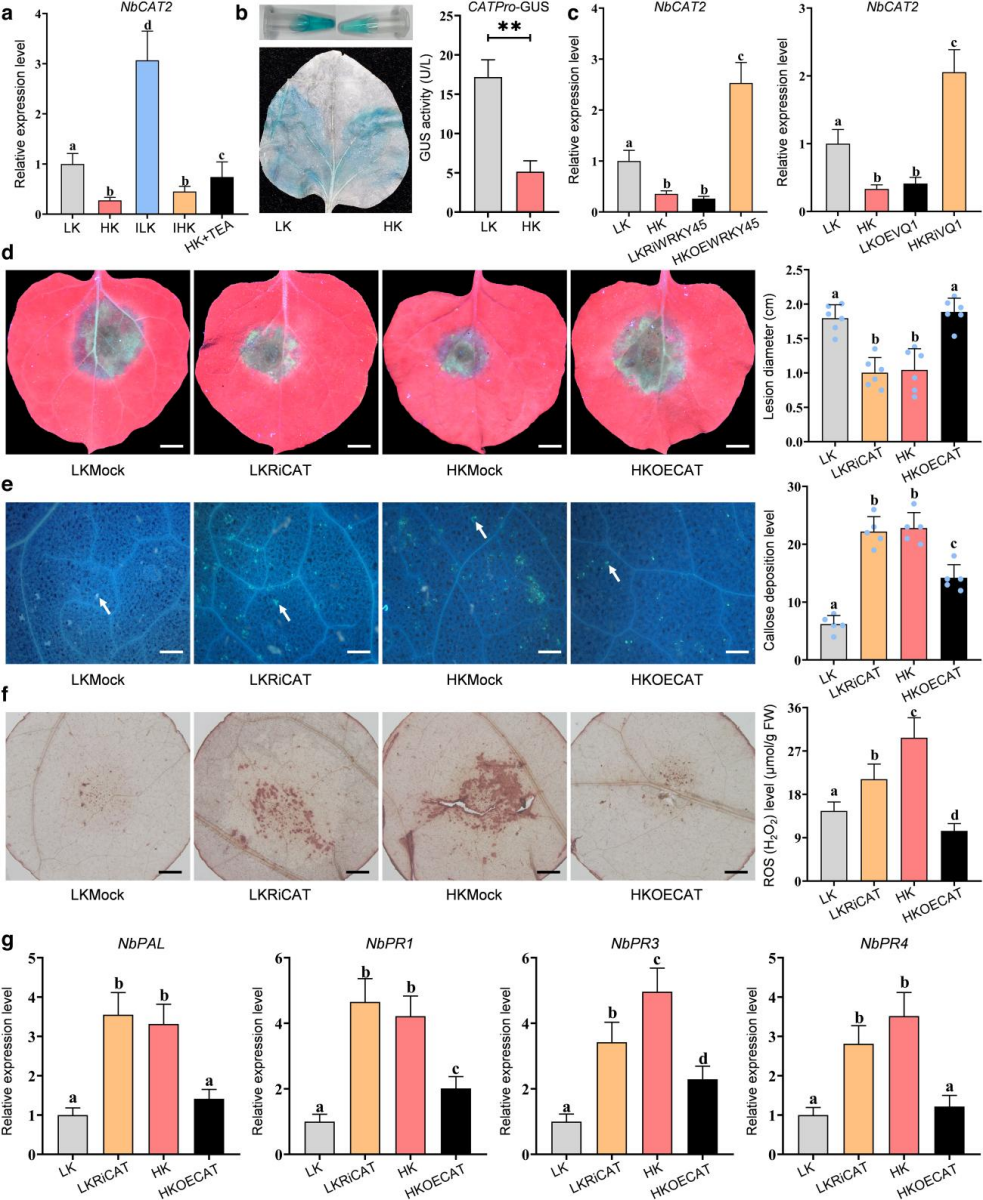
Suppression of *NbCAT2* expression increases ROS production and resistance of *N. benthamiana* to *P. parasitica* under HK conditions. a) Determination of *NbCAT2* expression in HK and LK *N. benthamiana* following *P. parasitica* infection; *n* = 3. b) GUS activity assay showing the responsiveness of *NbCAT2* expression in *N. benthamiana* to K supplementation; *n* = 3. c) RT-qPCR analysis of *NbCAT2* expression regulated by NbWRKY45 and NbVQ1 in HK and LK *N. benthamiana*; *n* = 3. d) Lesion phenotypes of HK and LK *N. benthamiana* with *NbCAT2* overexpression or RNAi-mediated silencing; *n* = 6. e) *NbCAT2* overexpression suppressed *P. parasitica*–induced callose deposition in HK and LK *N. benthamiana*; bar = 50 *μ*m; *n* = 5. f) DAB staining assay showing *P. parasitica*-triggered ROS (H_2_O_2_) accumulation in HK and LK *N. benthamiana* with *NbCAT2* silencing; bar = 100 mm; *n* = 3. g) *NbCAT2* upregulation suppressed the expression of resistance-related genes in HK and LK *N. benthamiana* following *P. parasitica* infection; *n* = 3. Data are presented as the mean ± Sd. Different letters and “**” represent significant differences based on *P* < 0.05. Statistical analysis in a) and c to g) is determined by 1-way ANOVA followed by post hoc Tukey test, and Student's *t*-test in b).

This study indicated that K addition suppressed *NbCAT2* expression, whereas *P. parasitica* induced its upregulation in *N. benthamiana*, particularly under LK conditions (Fig. 6a). A decrease in cellular K content caused by TEA treatment resulted in *NbCAT2* upregulation, and the downregulation of *NbCAT2* caused by adding K content was dose-dependent in *N. benthamiana* (Figs. 6a and S2b). GUS staining further confirmed the repression of *NbCAT2* expression by K supplementation (Fig. 6b). Meanwhile, *NbCAT2* expression was activated by NbWRKY45 in LK *N. benthamiana* but suppressed by NbVQ1 in HK *N. benthamiana* (Fig. 6c). To further examine the function of NbCAT2 in mediating resistance under different K conditions, transgenic LK and HK *N. benthamiana* lines with stable *NbCAT2* overexpression or RNAi-mediated silencing (LK/HKOECAT2 and LK/HKRiCAT2; Fig. S11a to d) were generated. The silencing of *NbCAT2* promoted callose deposition and ROS production, thereby enhancing the resistance in LK *N. benthamiana* to both *P. parasitica* and *B. cinerea* (Figs. 6d to f and S11a to e). In parallel, the expression levels of resistance-related genes, including *NbPAL*, *NbPR1*, *NbPR3*, and *NbPR4*, were upregulated in LK *N. benthamiana* following *NbCAT2* silencing (Fig. 6g). In contrast, *P. parasitica*–induced callose deposition and ROS production in HKOECAT2 plants were lower than those in HKMock (Fig. 6e and f). Furthermore, *NbCAT2* overexpression suppressed the expression of *NbPAL*, *NbPR1*, *NbPR3*, and *NbPR4*, which caused a decrease in resistance of HKOECAT2 plants against both pathogens compared with HKMock (Figs. 6d and g and S11a to e). These results suggest that NbWRKY45-activated *NbCAT2* expression negatively regulates ROS production and the associated resistance in *N. benthamiana* under LK conditions.

### NbVQ1 inhibits NbWRKY45 binding to the *NbCAT2* promoter to enhance resistance in *N. benthamiana* under HK conditions

**Figure 7:**
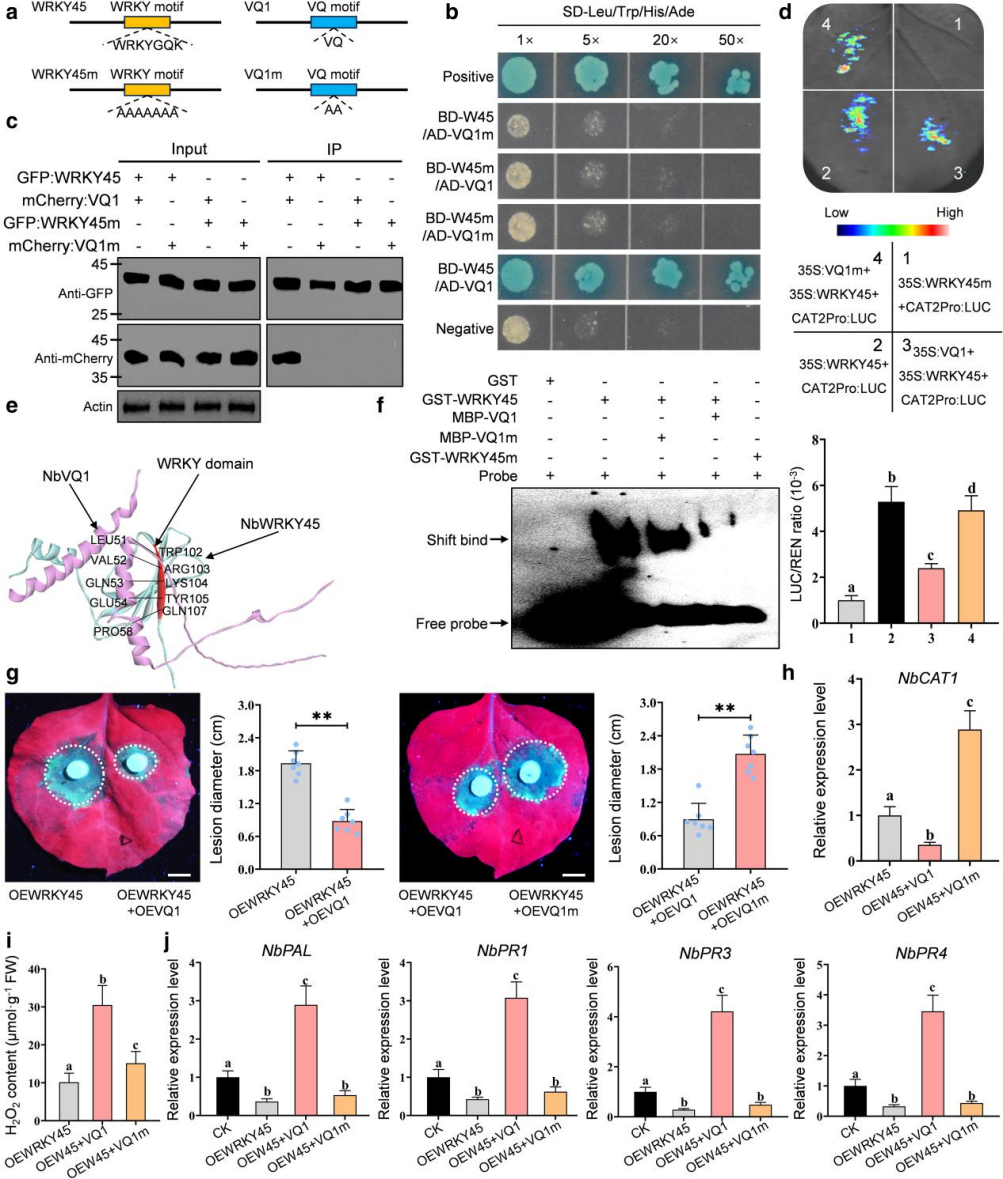
Interaction with NbVQ1 suppresses the binding affinity of NbWRKY45 to the *NbCAT2* promoter. A) Schematic representation of mutation models for NbWRKY45 and NbVQ1. The residue motifs of NbWRKY45 and NbVQ1 that are responsible for their interaction are mutated into Ala and generate NbWRKY45 m and NbVQ1m. b) Y2H assay identifying the hub motifs responsible for NbVQ1-NbWRKY45 interaction. c) Co-IP assay confirming that the VQ motif in NbVQ1 and the WRKYQK motif in NbWRKY45 mediate NbVQ1-NbWRKY45 interaction. d) LUC reporter assay evaluating the binding affinity of NbWRKY45 and NbWRKY45m to the *NbCAT2* promoter in the presence of *NbVQ1* or its mutant *NbVQ1m* in *N. benthamiana*. LUC activity assay shows that the presence of NbVQ1 suppresses the binding activity of NbWRKY45 and NbWRKY45m to the *NbCAT2* promoter in planta; *n* = 3. e) AlphaFold displaying the interaction model of NbWRKY45 and NbVQ1. f) EMSA assay showing the inhibitory effect of *NbVQ1* on *NbWRKY45* binding to the *NbCAT2* promoter. Mutations in WRKYQK motif result in loss of binding affinity of NbWRKY45 to *NbCAT2* promoter. g) Co-overexpression of *NbWRKY45* and *NbVQ1* or *NbVQ1m* in *N. benthamiana* resistance to *P. parasitica* revealed that suppression of NbWRKY45-mediated negative regulation of resistance depends on its interaction with *NbVQ1*. The effect of NbVQ1 in suppressing NbWRKY45 function that negatively regulates *N. benthamiana* resistance depends on their interaction; *n* = 7. h and j) Expression levels of *NbCAT2* and resistance-related genes in *N. benthamiana* with *NbWRKY45*, *NbWRKY45*-*NbVQ1*, or *NbVQ1m* overexpression; *n* = 3. i) NbVQ1 suppresses NbWRKY45-mediated reduction in ROS (H_2_O_2_) content in *N. benthamiana* following *P. parasitica* infection; *n* = 3. Data are presented as the mean ± Sd. Different letters and “**” represent significant differences based on *P* < 0.05. Statistical analysis in d) and h to j) is determined by 1-way ANOVA followed by post hoc Tukey test, and Student's *t*-test in g).

To elucidate the mechanisms by which the NbVQ1-NbWRKY45 complex mediated *NbCAT2* expression and resistance in *N. benthamiana* under different K conditions, the key amino acid residues of NbWRKY45 and NbVQ1 required for their interaction were identified using AlphaFold 3.0 (Fig. 7e). Further Y2H assays demonstrated that the mutation of VQ to AA in NbVQ1 (NbVQ1m) abolished its interaction with NbWRKY45, whereas the mutation of the WRKYGQK motif in NbWRKY45 (NbWRKY45m) disrupted its binding to NbVQ1 (Fig. 7a, b, and e). Co-IP assays further confirmed that the VQ motif of NbVQ1 and the WRKYGQK motif of NbWRKY45 were essential for their in vivo interaction (Fig. 7a, c, and e). Subsequent LUC reporter and EMSA assays revealed that NbWRKY45m lost its promoter-binding capacity with *NbCAT2*, underscoring the importance of these residues for binding affinity (Fig. 7d and f). Notably, NbVQ1 was observed to inhibit the binding of NbWRKY45 to the *NbCAT2* promoter, whereas NbVQ1m lost this regulatory function, demonstrating the dependency of NbVQ1 in inhibiting NbWRKY45 function on their interaction (Fig. 7d and f). Moreover, the FoldX prediction confirmed that the NbVQ1 interaction decreased the promoter-binding affinity of NbWRKY45 to *NbCAT2* (Table S15).

Subsequently, the role of the NbVQ1-NbWRKY45 complex in regulating the resistance of *N. benthamiana* to *P. parasitica* was investigated (Fig. 7g to j). Lesion diameters in plants coexpressing *NbVQ1* and *NbWRKY45* (OEWV) were significantly smaller than those in plants overexpressing *NbWRKY45* alone (OEWRKY45) (Fig. 7g). The presence of NbVQ1 alleviated the NbWRKY45-induced *NbCAT2* upregulation and promoted ROS production following *P. parasitica* infection (Fig. 7h and i). Furthermore, the expressions of resistance genes, including *NbPAL*, *NbPR3*, *NbPR4*, and *NbPR1*, were markedly higher in the OEWV plants than in OEWRKY45 (Fig. 7j). These results indicated that NbVQ1 attenuates the regulatory function of NbWRKY45 in suppressing basal immunity in *N. benthamiana*. Subsequently, *P. parasitica* was inoculated into *N. benthamiana* plants coexpressing *NbWRKY45* and mutated *NbVQ1 m* (OEWVm). In these plants, lesion diameters were significantly larger than those in the OEWV plants, which correlated with elevated *NbCAT2* expression (Fig. 7g and h). Moreover, the expression of resistance-related genes and the levels of ROS production were markedly lower in OEWVm than in OEWV, demonstrating that the ability of NbVQ1 to suppress NbWRKY45 function was dependent on their physical interaction (Fig. 7i and j). Overall, these findings indicate that NbVQ1 inhibits the binding affinity of NbWRKY45 to the *NbCAT2* promoter, thereby reducing *NbCAT2* transcription, promoting ROS accumulation, and ultimately enhancing resistance in *N. benthamiana* under HK conditions.

### Potassium supplementation promotes NbVQ1-NbWRKY45 interaction to enhance resistance in *N. benthamiana*

**Figure 8:**
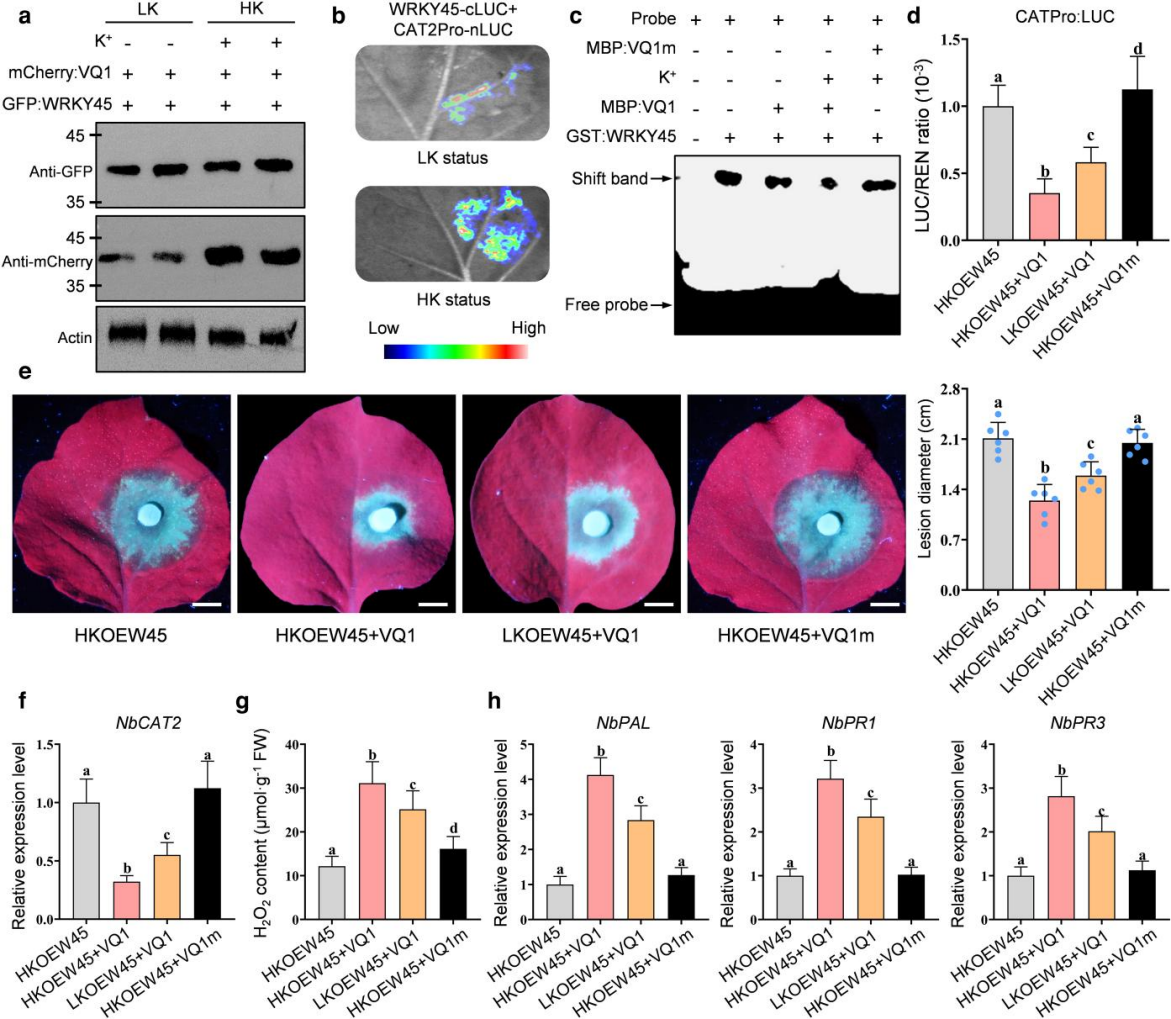
NbVQ1 more effectively suppresses NbWRKY45 function to enhance *N. benthamiana* resistance under HK conditions. a) Co-IP assay detecting the interaction between NbVQ1 and NbWRKY45 under HK and LK conditions. b) Dual-LUC reporter assay assessing the interaction level between NbVQ1 and NbWRKY45 under HK and LK conditions. c) EMSA assay showing the effect of NbVQ1 on NbWRKY45 binding affinity to the *NbCAT2* promoter under HK and LK conditions. Adding K^+^ promotes the effects of NbVQ1 in reducing binding affinity of NbWRKY45 to *NbCAT2* promoter in vitro. d) LUC activity assay evaluating NbWRKY45 binding to the *NbCAT2* promoter in the presence of NbVQ1 or its mutant NbVQ1m in HK *N. benthamiana*. The effect of NbVQ1 in reducing binding affinity of NbWRKY45 to *NbCAT2* promoter in HK *N. benthamiana* is higher than that in LK *N. benthamiana*, while NbVQ1m loses this function even under HK condition; *n* = 3. e) Lesion diameters of HK and LK *N. benthamiana* overexpressing *NbWRKY45*, *NbWRKY45*-*NbVQ1*, or *NbWRKY45*-*NbVQ1m*; *n* = 6. f and h) Expression levels of *NbCAT2* and resistance-related genes in HK and LK *N. benthamiana* overexpressing *NbWRKY45*, *NbWRKY45*-*NbVQ1*, and *NbWRKY45*-*NbVQ1m*; *n* = 3. g) *P. parasitica*–induced ROS (H_2_O_2_) accumulation in HK and LK *N. benthamiana* overexpressing *NbWRKY45*, *NbWRKY45*-*NbVQ1*, or *NbWRKY45*-*NbVQ1m*; *n* = 3. Data are presented as the mean ± Sd. Different letters represent significant differences at *P* < 0.05 based on 1-way ANOVA followed by post hoc Tukey test.

Here, the effects of K supplementation on the NbVQ1-NbWRKY45 complex were further examined. Then, AlphaFold 3.0 and FoldX analyses indicated that the presence of K^+^ enhanced the interaction affinity between NbVQ1 and NbWRKY45 (Table S15). Further LUC reporter and Co-IP assays demonstrated that the interaction of NbVQ1 with NbWRKY45 in HK *N. benthamiana* was stronger than that in LK plants (Fig. 8a and b). Consistently, pull-down results confirmed that adding K^+^ observably promoted the interaction between NbVQ1 and NbWRKY45 in vitro (Fig. S12). In addition, EMSA and LUC assays revealed that the inhibitory effect of NbVQ1 on the binding affinity of NbWRKY45 to the *NbCAT2* promoter was more pronounced under HK conditions (Fig. 8c and d). In contrast, the NbVQ1m mutant failed to prevent NbWRKY45 from binding to *NbCAT2* promoter, even under HK conditions (Fig. 8c and d), suggesting that K addition suppressed *NbCAT2* expression by promoting the interaction between NbVQ1 and NbWRKY45.

Furthermore, NbVQ1 reduced the ability of NbWRKY45 to trigger *NbCAT2* expression and interfered with resistance in *N. benthamiana* under both HK and LK conditions (Fig. 8e and f). Although NbVQ1 suppressed NbWRKY45-related functions under LK conditions, its inhibitory effects were more significant in HK plants (Fig. 8e and f). The ROS content and expression levels of resistance genes in LKOEWV plants were significantly higher than those in HKOEWRKY45 but lower than those in HKOEWV following pathogen challenge, supporting that adding K enhanced NbVQ1-NbWRKY45 function to promote plant basal immunity (Fig. 8g and h). Notably, lesion diameters in HKOEWVm plants were larger than those in both HKOEWV and LKOEWV (Fig. 8e). In parallel, *NbCAT2* expression was upregulated in HKOEWVm plants, resulting in reduced ROS levels compared with those in HKOEWV and LKOEWV plants (Fig. 8f and g). The upregulation of resistance genes in HK *N. benthamiana* was also suppressed in *NbVQ1m-NbWRKY45* coexpression lines, demonstrating the dependency of K-enhanced resistance on NbVQ1-NbWRKY45 interaction (Fig. 8h). Collectively, supplementation of K^+^ facilitated the NbVQ1-NbWRKY45 interaction and enhanced NbVQ1-mediated inhibition of NbWRKY45 transactivation on *NbCAT2*, thereby promoting ROS burst and basal resistance in HK *N. benthamiana*.

### Loss function of NbWRKY45 facilitates plant drought tolerance

WRKYs have been shown to participate in plant resistance to abiotic stresses; for example, AtWRKY75 plays a role in drought response (Lim et al. 2022). Given the sequence homology between NbWRKY45 and AtWRKY75, we hypothesized that NbWRKY45 might also mediate drought tolerance in *N. benthamiana* (Fig. S7). Typically, *NbWRKY45* was upregulated only at the early stage of drought treatment, and its silencing significantly enhanced tolerance of transgenic *N. benthamiana* seedlings (RiWRKY45) to drought stress (Fig. S13a and b). Mechanistically, although *NbWRKY45* silencing initially promoted early H_2_O_2_ accumulation, it subsequently caused more significant decreases in H_2_O_2_ content in *N. benthamiana* under drought stress (Fig. S13c). This reduction in late-stage ROS was accompanied by a marked increase in superoxide dismutase (SOD) activity, while catalase (CAT) activity remained unchanged, leading to a significant decrease in drought-induced malondialdehyde (MDA) accumulation (Fig. S13e to h). Moreover, RiWRKY45 seedlings exhibited significantly higher water, flavonoid, and proline contents compared with wild type (WT/Mock) plants under drought stress, supporting that NbWRKY45 negatively regulated plant drought tolerance (Fig. S13d to g). It is well established that ROS signals act as key mediators of stomatal movement, thereby avoiding the reduction of water content and conferring plant tolerance to drought stress (Sierla et al. 2016; Qi et al. 2018; Yang et al. 2024). In line with this, our results showed that NbWRKY45 silencing promoted *NbRBOHD* expression and ROS production, which in turn led to smaller stomatal apertures under drought conditions (Fig. S13i and f). Consistently, we found that *NbCAT2* was also upregulated at the early stage of drought treatment, while its silencing also promoted early H_2_O_2_ production and increased water content in *N. benthamiana* under drought conditions (Fig. S14a to c). Notably, *NbCAT2* silencing did not affect the increases in CAT and SOD activity, leading to decreased MDA accumulation in *N. benthamiana* under drought stress, which was explained by the upregulations of other 4 *NbCATs* in *N. benthamiana* at later stage of drought treatment (Fig. S14a to e). This suggested that NbCAT2 was not responsible for scavenging drought-related oxidative stress, and might participate in regulating the early ROS signaling under drought stress. Consistently, *NbCAT2* silencing also induced stomatal closure in *N. benthamiana* under drought stress (Fig. S14f). These results suggested that NbWRKY45-NbCAT2 axis might involve in plant drought tolerance via regulating ROS-related stomatal apertures.

Subsequently, we performed ChIP-seq to identify the potential targets of NbWRKY45. The results revealed that NbWRKY45 bound not only to *NbCAT2* promoter, but also interacted with promoters of numerous genes involved in flavonoid biosynthesis, including *NbFG3*, *Nb4CL*, and *NbUGT72B1* (Fig. S15a). Notably, these genes encoded synthesis of various flavonoids with antioxidant activity, especially kaempferol-glucoside (Du et al. 2024a). Further, transient overexpression and LUC reporter assays confirmed that NbWRKY45 transactivated these genes by directly binding to their promoters in vivo (Fig. S15b and c). Functional analysis showed that overexpression of *NbFG3* or *NbUGT72B1* reduced the capacity of *N. benthamiana* extracts in scavenging ABTS (2,2′-Azino-bis(3-ethylbenzothiazoline-6-sulfonic acid) diammonium salt) radical (Fig. S15d). Together, these findings suggested that NbWRKY45 might also reprogram the flux of the flavonoid biosynthesis to reduce antioxidant flavonoids, ultimately decreased plant drought tolerance.

### Activation of basal resistance by VQ-WRKY-CAT is conserved among plants

**Figure 9:**
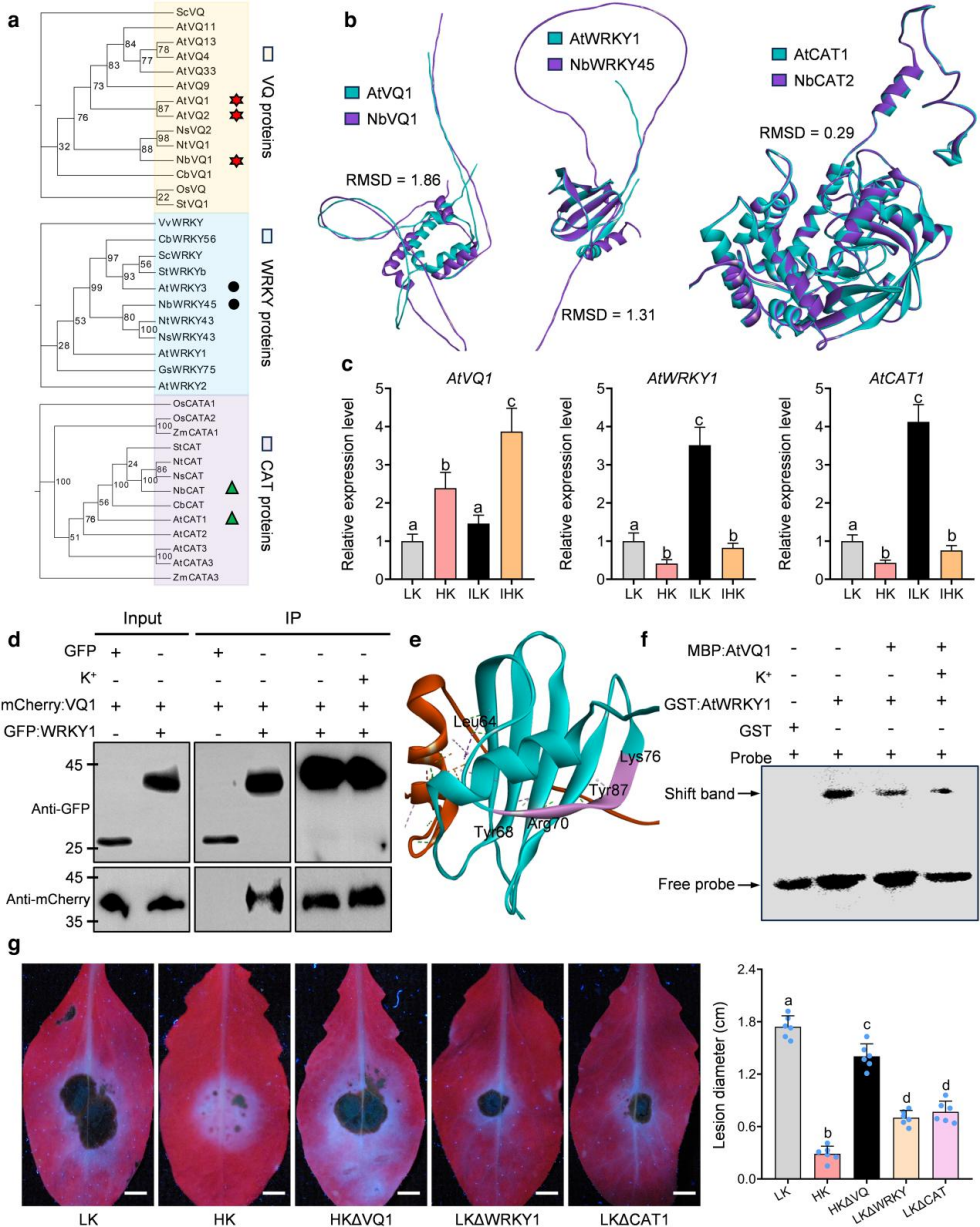
VQ-WRKY-CAT mediates conserved resistance mechanisms in *Arabidopsis thaliana* under different K conditions. a) Phylogenetic analysis of NbVQ1, NbWRKY45, NbCAT2, and their homologs from *A. thaliana* and others using ML algorithm based on bootstrap = 2,000. b) Structural comparisons exhibiting high similarity among NbVQ1, NbWRKY45, NbCAT2, and their *A. thaliana* homologs. The protein structures were predicted using AlphaFold 3.0. c) RT-qPCR analysis of *AtVQ1*, *AtWRKY1*, and *AtCAT1* expression in HK and LK *A. thaliana* following *B. cinerea* infection; *n* = 3. d) Co-IP assay confirming the enhancing effects of K^+^ supplementation on AtVQ1-AtWRKY1 interaction in vivo. e) Prediction of interface residues responsible for AtVQ1-AtWRKY1 interaction using AlphaFold modeling. f) EMSA assay showing that AtVQ1 suppresses AtWRKY1 binding affinity to the *AtCAT1* promoter under different K conditions. g) AtVQ1-AtWRKY1-AtCAT1 module mediates K-associated resistance in *A. thaliana* to *B. cinerea*; *n* = 6. Data are presented as means ± Sd. Different letters represent significant differences at *P* < 0.05 based on 1-way ANOVA followed by post hoc Tukey test.

Here, a maximum likelihood (ML) tree was constructed for NbVQ1, NbWRKY45, and NbCAT2, revealing that these proteins were conserved across most plant species and were homologous to AtVQ1, AtWRKY1, and AtCAT1 of *Arabidopsis thaliana* (Fig. 9a). Structural comparison and sequence alignment further indicated that AtVQ1, AtWRKY1, and AtCAT1 shared similar structures and conserved functional domains with their homologs in *N. benthamiana* (Figs. 9b and S16a and b). Meanwhile, K supplementation enhanced *AtVQ1* expression in HK *A. thaliana* following *B. cinerea* infection, whereas no significant induction of *AtVQ1* expression was observed in LK plants (Fig. 9c). In contrast, both *AtWRKY1* and *AtCAT1* were strongly upregulated in LK *A. thaliana* but downregulated in HK plants after *B. cinerea* infection (Fig. 9c). These findings suggest that AtVQ1-AtWRKY1-AtCAT1 module might mediate conserved mechanisms that regulate plant basal resistance under different K conditions.

Subsequently, AtVQ1 was found to physically interact with the WRKYGQK domain of AtWRKY1 in the nucleus, and this interaction was enhanced by increased in planta K levels (Figs. 9d and e and S16c). Furthermore, AtWRKY1 transactivated *AtCAT1* expression by binding to its promoter region, which contained the TTGACC cis-element (Figs. 9f and S16d and e; Table S13). Moreover, AtVQ1 suppressed the binding of AtWRKY1 to the *AtCAT1* promoter, and this suppression enhanced AtWRKY1 transactivation activity in the presence of K^+^ (Fig. 9f). The functional analysis demonstrated that AtVQ1-AtWRKY1-AtCAT1 played the critical roles in *A. thaliana* resistance under different K conditions. Specifically, *AtVQ1* knockout (ΔAtVQ1) significantly reduced the resistance of HK *A. thaliana* to *B. cinerea* compared with WT plants (Fig. 9g). In contrast, the knockout of both *AtWRKY1* and *AtCAT1* (ΔAtWRKY1 and ΔAtCAT1) increased the resistance of *A. thaliana* to *B. cinerea*, even under LK conditions (Fig. 9g). Collectively, these results suggest that the VQ-WRKY-CAT module represents a conserved mechanism that mediates plant basal resistance associated with in planta K nutrient status.

## Discussion

**Figure 10:**
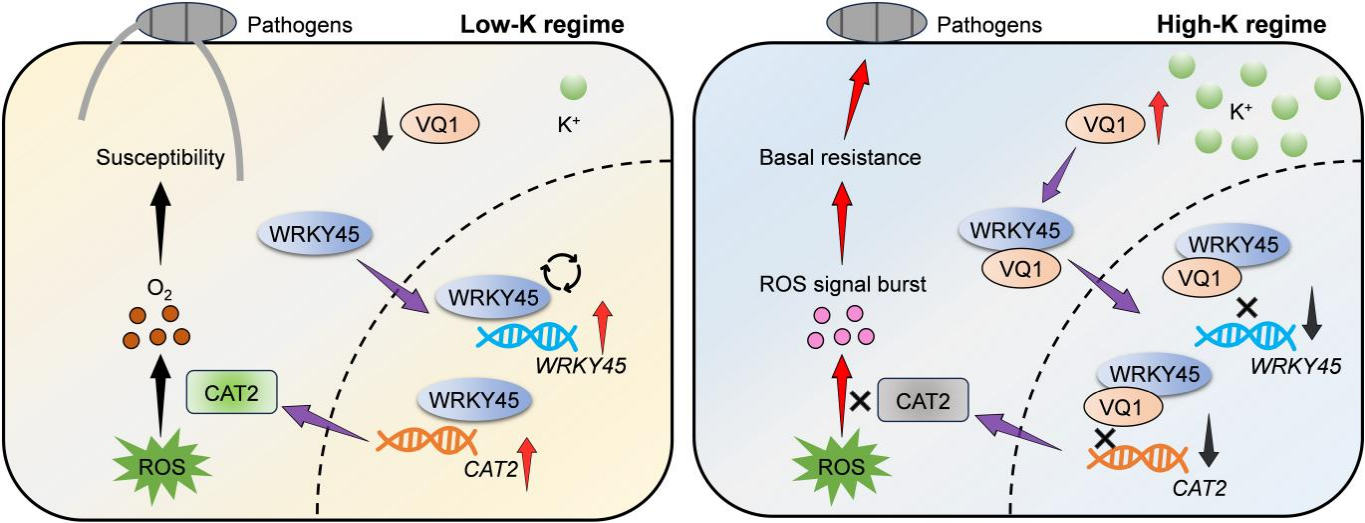
Schematic model of NbVQ1-NbWRKY45-NbCAT2 regulatory module mediating K-associated ROS burst and basal resistance in plants. The NbVQ1-NbWRKY45-NbCAT2 transcriptional module mediates ROS accumulation in response to *P*. *parasitica* infection in HK plants. Elevated K levels promote NbVQ1 expression and enhance its interaction with NbWRKY45, thereby disrupting NbWRKY45 binding to *NbCAT2* promoter. This suppression of *NbCAT2* transcription leads to increased ROS production and strengthened resistance of *N*. *benthamiana* to *P*. *parasitica*. Collectively, this study highlights the importance of the “plant nutrient immunity” concept in crop disease management through the optimization of in planta K nutrient status.

K is involved in numerous biological processes in plants and plays a critical role in enhancing resistance to diverse pathogens (Amtmann et al. 2008; Du et al. 2023b, 2024b). In the present study, a conserved mechanisms by which the VQ-WRKY-CAT2 module regulates ROS-related defense responses were systematically investigated in *N. benthamiana* and *A. thaliana* under HK conditions (Fig. 10). Elevated K levels promoted the pathogen-triggered activation of multiple resistance responses, especially ROS burst mediated by NbVQ1-NbWRKY45-NbCAT2 module in HK plants. NbWRKY45 formed a self-transactivation loop to trigger *NbCAT2* expression via its promoter binding affinity, then scavenged ROS and caused the reduced resistance in LK plants. Notably, NbVQ1 interacted with the WRKYGQK motif of NbWRKY45 via its VQ motif, thereby reducing the binding affinity of NbWRKY45 to the *NbCAT2* promoter, which suppressed ROS scavenging and enhanced resistance in *N. benthamiana*. Moreover, elevated K promoted the physical interaction between NbVQ1 and NbWRKY45, then enhanced the ability of NbVQ1 to suppress NbWRKY45 function in upregulating *NbCAT2*, leading to stronger ROS burst and enhanced resistance in HK *N. benthamiana*. These findings collectively reveal that K nutrition directly modulates the strength of VQ-WRKY-CAT regulatory node, providing a molecular basis for K-enhanced immunity. In addition, the suppression of NbWRKY45 might promote drought tolerance of *N. benthamiana* through regulating ROS-related stomatal movement and flavonoid biosynthesis. Overall, these findings can provide practical avenues for disease management through K fertilization and targeted breeding and then contribute to the theoretical frameworks of nutritional immunity and tradeoffs in stress responses.

### ROS homeostasis is essential for the broad-spectrum disease resistance of plant caused by adding K nutrient

Plants are constantly threatened by both natural pathogens and abiotic stresses (Ngou et al. 2022; Wu et al. 2023). To cope with these challenges, they have evolved a sophisticated immune system that includes calcium influx, activation of MAPKs, transcriptional reprogramming of defense-related genes, and ROS production (Ngou et al. 2021; Yuan et al. 2021). The activation of immune responses is associated with plant performance and largely determined by nutrient availability (Cheng et al. 2019; Luan and Wang 2021). Among the essential nutrients, K plays a particularly critical role in the resistance to pathogenic microbes, with elevated K levels conferring broad-spectrum protection (Amtmann et al. 2008; Du et al. 2023b, 2024b). Previous research has demonstrated that K can promote plant resistance through multiple processes, including enhanced accumulation of defensive metabolites, upregulation of resistance genes, and maintenance of membrane homeostasis (Du et al. 2023b, 2024b). In the present study, sufficient K (HK) status was found to significantly potentiate the plant basal immunity via accelerating activation of key defense mechanisms, including upregulation of resistance genes, extensive callose deposition, defensive metabolite production, and, most critically, a robust ROS burst in HK plants. As a central component of plant immune signaling, ROS has increasingly been recognized as a small-molecule secondary messenger that orchestrates resistance responses (Mittler et al. 2022; Wu et al. 2023). Generally, transient ROS burst activates signal transduction pathways, whereas its prolonged accumulation disrupts cellular redox homeostasis and causes oxidative damage (Mittler 2017; Wu et al. 2023). Increasing evidence indicates that K may crosstalk with ROS production in the regulation of plant growth and defense responses (Liu et al. 2022; Luo et al. 2022). Our data indicate that HK plants exhibited a markedly stronger ROS burst upon *P. parasitica* infection compared with LK plants, correlating with higher expression of *NbRBOHF* and especially *NbRBOHD* that responsible for producing ROS burst in plants under HK status. ROS signaling in plants orchestrates diverse resistance events that protect against pathogens, including the synthesis of defensive metabolites, induction of programmed cell death, and upregulation of resistance genes (Qi et al. 2017; Wang et al. 2024a). Here, in HK *N. benthamiana*, ROS-mediated resistance pathways, such as resistance gene activation and defensive metabolite accumulation, were significantly stronger than those in LK plants during *P*. *parasitica* challenge. Considering the role of ROS burst and its downstream resistance pathways in mediating the broad-spectrum resistance of plants (Waszczak et al. 2018; Wang et al. 2024a), the elevated ROS burst observed in HK plants can provide a mechanistic explanation of how K nutrition enhances resistance to a wide range of pathogens. Beyond immune regulation, ROS generation also plays a critical role in defense against abiotic stresses, including heat, salinity, and UV irradiation (Ravi et al. 2023; Wang et al. 2024b). In this study, the ROS homeostasis was further shown to mediate the drought tolerance in *N*. *benthamiana* through the regulation of stomatal movement. Similarly, it was found that ROS functions as a central hub in controlling stomatal dynamics, with ROS burst promoting stomatal closure, thereby enhancing drought tolerance (Gao et al. 2022). Given the involvement of K in regulating ROS burst, optimal K supplementation might also improve plant drought tolerance. Consistently, changes in K availability have been reported to determine plant resistance to multiple abiotic stresses, including salinity, drought, and osmotic stress (Mostofa et al. 2022; Li et al. 2023; Feng et al. 2025). These results support potential application value of optimal K nutrition supplementation in safeguarding crops against the detrimental effects of both biotic and abiotic stresses in practice. Although the specific role of ROS in these processes remains to be fully elucidated, our present results prove that K nutrition and associated ROS homeostasis are important for plant resistance to biotic and abiotic stresses, which holds substantial significance for the sustainable development of agriculture.

### Potassium supplementation deploys NbVQ1 to turn off NbWRKY45-NbCAT module for promoting ROS burst in plants under biotic stress

ROS have traditionally been regarded as metabolic by-products that are harmful to exposed cells and tissues (Mittler et al. 2022; Wu et al. 2023). Thus, plants have evolved complex regulatory systems to maintain ROS homeostasis through the coordinated interplay among ROS production, scavenging, and transport (Mittler et al. 2022). In this study, the K nutritional status of plants also influenced ROS burst, with sufficient K supply promoting a stronger ROS burst in response to pathogen attack. This observation aligns with earlier reports that in planta K nutrient level affects ROS production, then this K-associated signal could act as a metabolic switch, stimulating catabolic processes and redirecting energy toward adaptation (Demidchik et al. 2003, 2010; Shabala et al. 2014; Shabala 2017). In plants, ROS signals were primarily generated by RBOHs, which catalyzed the reduction of oxygen to superoxide and subsequently converted to H_2_O_2_ in the apoplast (Wang et al. 2020). To mitigate oxidative stress, multiple antioxidant enzymes, including ascorbate peroxidase (APX1), CAT, thylakoid aperoxidase, alternative oxidase in mitochondria, and Cu-Zn-superoxide dismutase 2, are activated to scavenge excess ROS and protect plant tissues from damage (Qi et al. 2017). Among these, CAT plays a central role in regulating intracellular H_2_O_2_ levels and maintaining redox balance, thereby preventing oxidative injury caused by excessive ROS accumulation (Willekens et al. 1997; Apel and Hirt 2004). We found that *NbCAT2* was highly expressed in LK *N*. *benthamiana* plants, where it actively scavenged ROS during *P*. *parasitica* infection, thereby dampening defense. WRKY transcription factors are known as the regulators of *CAT* expression, functioning either directly or indirectly in response to pathogen invasion and abiotic stress (Jiang et al. 2017; Javed et al. 2023). At the core of K-enhanced resistance lies the NbVQ1-NbWRKY45-NbCAT2 module, which functions as a molecular switch controlling ROS homeostasis. Under LK conditions, NbWRKY45 activated its own expression through a self-regulatory loop and directly transactivated *NbCAT2*, thereby suppressing ROS accumulation and compromising basal defense, which rendered *N. benthamiana* more susceptible to pathogen attack. This interaction impedes NbWRKY45 binding to the *NbCAT2* promoter, reducing *NbCAT2* transcription and thereby enhancing the pathogen-triggered ROS burst and resistance in HK plants. In contrast, elevated K levels induced NbVQ1 expression, enabling its physical interaction with NbWRKY45 at the WRKY motif. This interaction impeded NbWRKY45 binding to the *NbCAT2* promoter, reducing *NbCAT2* transcription, thereby enhancing pathogen-triggered ROS burst and resistance in HK plants. Recent studies have shown that VQ proteins (characterized by the FxxxVQxLTG motif) act as cofactors that interact with WRKYs to regulate plant growth and defense (Ma et al. 2023; Dong et al. 2024; Huang et al. 2025). Although VQ proteins have often been shown to promote WRKY function, several reports have demonstrated that VQs can also suppress WRKY transcriptional activity via physical interactions (Dong et al. 2024). For instance, MdVQ37 interacts with MdWRKY100, repressing its transcriptional activity and thereby reducing apple resistance to *Colletotrichum fructicola* (Dong et al. 2024). Consistent with this, our results revealed that the NbVQ1 suppressed NbWRKY45-mediated *NbCAT2*, thereby promoting ROS burst under HK status. Importantly, increasing K content promoted the interaction of NbVQ1 with NbWRKY45, which further amplified NbVQ1-mediated suppression of NbWRKY45 function. This K-dependent enhancement suggests that in planta K levels might be transduced into a regulatory signal through this module, although the precise biochemical mechanism (eg, direct K^+^ binding or conformational changes) remains to be elucidated.

Beyond biotic resistance, numerous studies showed that VQ-WRKY module exerts negative or positive function in regulating plant tolerance to abiotic stress (Cheng et al. 2012). For instance, Duan et al. (2025) recently demonstrated that the MdVQ17/MdVQ37 suppressed the promoter binding ability of MdWRKY100 to suppress *MdAKT1* and *MdPER57* expression, then negatively regulated salt tolerance via increasing Na^+^/K^+^ ratio and reducing ROS scavenging capacity in apple. Here, we identified a parallel role for the NbWRKY45-NbCAT2 axis in negatively regulating drought tolerance through controlling stomatal aperture. Silencing *NbWRKY45* or *NbCAT2* promoted stomatal closure and enhanced drought survival, indicating that their ROS-scavenging activity is critical for maintaining stomatal opening. Despite the mechanistic similarity that VQ suppresses WRKY function in both modules, NbVQ1-mediated inhibition of NbWRKY45 enhances plant disease resistance under HK conditions, whereas MdVQ17/MdVQ37-mediated suppression of MdWRKY100 reduces plant salt tolerance. These phenomena establish the functional diversity of VQ-WRKY modules that have evolved in the context of regulating plant ROS homeostasis and defending against various stresses. And, the contrasting outcomes likely reflect differences in stress type (salt stress vs. pathogen infection) and nutritional context, highlighting the context-dependent plasticity of VQ-WRKY regulatory modules. Taken together, the VQ-WRKY-CAT regulatory module appears to play a dual role in both pathogen defense and abiotic stress tolerance, underscoring its function as a sophisticated signaling hub that enables plants to balance resistance and resource allocation under fluctuating environmental conditions. Moreover, conservation of this module across species indicates a universal strategy for engineering broad-spectrum resistance in future.

In summary, this study established a direct molecular connection between mineral nutrition and plant immunity, offering a compelling explanation for the disease-protective effects of K fertilization (Fig. 10). And, these findings provide a molecular framework that substantiates long-standing agronomic observations linking K fertilization with reduced disease incidence, thereby transforming the concept of “nutritional immunity” from a physiological correlation into a mechanistic reality.

## Materials and methods

### Plant growth conditions and drought treatments

*N. benthamiana* plants were cultivated in growth chambers maintained at 25 °C with 70% relative humidity and a 16-h light/8-h dark photoperiod. High- and low-potassium treatments were performed according to previously described protocols (Du et al. 2024b). To simulate drought stress, plants were treated with 10% (w/v) polyethylene glycol 6000 (PEG6000), and phenotypic changes were assessed after 10 d. There were at least 3 biological replicates per treatment, and the experiment was repeated 3 times.

### Pathogen inoculation assay

*P. parasitica* was reactivated on potato dextrose agar at 25 °C. Mycelial plugs were excised and inoculated onto the abaxial surface of detached *N. benthamiana* leaves. Inoculated leaves were placed on moist paper towels and incubated at 100% relative humidity for 3∼5 d. There were at least 3 biological replicates per treatment, and the experiment was repeated 3 times.

### *Agrobacterium*-mediated transformation and vector construction

*Agrobacterium tumefaciens* strain GV3101 carrying recombinant constructs was used for transient gene expression and VIGS in *N. benthamiana* leaves. Vector assembly was performed using the Basic Seamless Cloning and Assembly Kit (CU201-02, TransGen Biotech). A complete list of primers used in this study was provided in Table S14.

### Histochemical and quantitative ROS detection

For histochemical staining, leaves were incubated in 10 mg/mL 3,3′-diaminobenzidine (DAB) solution to detect ROS (H_2_O_2_) level, respectively, followed by destaining in ethanol. Callose deposition was visualized using 0.1% (v/v) aniline blue, also followed by ethanol destaining. Treated tissues were imaged under a light microscope. The relative intensity of ROS (H_2_O_2_) signals was quantified using a microplate reader as described by Jia et al. (2025), and absolute H_2_O_2_ levels were measured with a commercial assay kit (Beyotime Biotechnology, Shanghai, China). There were at least 3 replicates per treatment, and the experiment was repeated 3 times.

### GUS analysis

To evaluate promoter activity under HK conditions, the *NbCAT2* promoter was inserted into the pBI121 vector in place of the native 35S CaMV promoter to drive GUS expression. The resulting construct was introduced into *A. tumefaciens* GV3101. *N. benthamiana* leaves received a split-leaf pretreatment: the left half was infiltrated with sterile water (control), and the right half was infiltrated with 50 mm KCl for 12 h to create HK conditions. Thereafter, the *Agrobacterium* suspension harboring the CATpro-GUS construct was infiltrated into both halves. Following a suitable incubation period, GUS activity was assessed and visualized using a commercial GUS assay kit (Coolaber, Beijing, China).

### Construction of the transgenic *N. benthamiana* plants

To investigate the functions of NbCAT2, NbVQ1, and NbWRKY45, we deployed *Agrobacterium*-mediated transformation of *N. benthamiana* calli to construct the transgenic *N. benthamiana* with stable overexpression of representative genes. The positive overexpression vectors containing related genes were transformed into *A. tumefaciens* strain LBA4404, and positive clones were identified after sequencing and resistance screening. Then, the *A*. *tumefaciens* containing positive overexpression vectors were cocultivated with *N. benthamiana* calli on MS medium. Then, the positive transgenic calli were screened based on kanamycin resistance and further identified by RT-qPCR and western blot. After differentiation, budding and rooting on rooting MS medium, the T3 generation was obtained and verified using RT-qPCR and western blot.

Transgenic tobacco lines for RNAi silencing were generated via *A. tumefaciens*–mediated transformation using the pUCCRNAi vector. Surface-sterilized leaf patches from 4-wk-old plants were inoculated with an *A. tumefaciens* suspension (OD_600_ = 0.6) for 20 min in the dark with a shaker at 28 °C, 90 rpm. Followed by a 2-d cocultivation, leaf patches were transferred to a selection MS medium with 0.16 mg/L 6-benzylaminopurine (6-BA), 0.1 mg/L 1-naphthaleneacetic acid (NAA), 50 mg/L hygromycin, and 400 mg/L timentin to regenerate shoots from calli. Subsequently, Regenerated shoots were excised and subcultured on the same selection medium. Transgenic plants were validated by PCR at a genomic level. Then, the transgenic seedlings were subcultured on 2 and 0.5 K MS media. Fifteen days after culturing, the K content in representative seedlings was determined and used for pathogen inoculation assay. All primers were listed in Table S14.

### Yeast 1-hybrid and yeast 2-hybrid assays

The yeast 1-hybrid (Y1H) assay was performed as previously described by Du et al. (2024a). Promoter fragments (∼2,000 bp upstream of the start codon) of *NbWRKY45* and *NbCAT2* were amplified and inserted into the pHIS2 vector. The full-length cDNA of *NbWRKY45* was cloned into the pGADT7 activation domain vector. The resulting constructs were cotransformed into Y1HGold yeast strain and grown on SD/-Leu medium supplemented with 30 mm 3-amino-1,2,4-triazole (3-AT) at 30 °C for 3 d.

For the yeast 2-hybrid (Y2H) assay, the coding sequence of *NbVQ1* was cloned into the GAL4 DNA-binding domain vector (pGBKT7). The corresponding prey vector was cotransformed into the Y2HGold strain, and transformants were selected on SD/-Leu/-Trp/-His/-Ade medium containing 150 ng/mL aureobasidin A (AbA) at 30 °C for 3 to 5 d. All primers were listed in Table S14.

### LUC reporter assay

For the dual-LUC reporter assay, promoter regions of candidate genes were cloned into the pGreenII 0800-LUC vector (referred to as pLUC), and coding sequences of transcription factors were inserted into the pGreenII 62-SK vector (referred to as pSK). *A. tumefaciens* strain GV3101 carrying the respective constructs was cultured overnight and resuspended in infiltration buffer containing 10 mm MgCl_2_, 0.1 mm acetosyringone, and 20 mm 2-morpholinoethanesulphonic acid (MES) (pH 5.6). The bacterial suspension was adjusted to an OD_600_ of 0.6∼0.8. Equal volumes of pLUC and pSK *Agrobacterium* cultures were mixed and infiltrated into fully expanded leaves of 4∼6-wk-old *N*. *benthamiana* plants using a needleless syringe. After 48 h, the infiltrated leaves were sprayed with 0.1 m luciferin solution and incubated in the dark for 7 min. LUC signals were captured using a low-light cooled charge coupled device (CCD) imaging system (Lumazone Pylon 2048B, Princeton Instruments), and quantitative luminescence intensity was measured using a microplate reader. Each assay was performed at least 3 times. All primers were listed in Table S14.

### Co-IP assay

Full-length coding sequences of *NbWRKY45* and *NbVQ1* were fused with GFP and mCherry tags, respectively, under the control of the CaMV 35S promoter and cloned into a binary vector. *A*. *tumefaciens* GV3101 strains harboring the respective constructs were coinfiltrated into fully expanded young leaves of *N*. *benthamiana* at an OD_600_ of 0.5. Samples expressing GFP and *NbVQ1*-mCherry were included as control. At 48 h postinfiltration, leaf tissues displaying GFP and mCherry fluorescence were harvested and flash-frozen in liquid nitrogen. Total proteins were extracted using radioimmunoprecipitation assay buffer according to standard laboratory protocols. Protein extracts were incubated overnight at 4 °C with GFP-Trap agarose beads under gentle rotation. After washing, bead-bound proteins were eluted in SDS-PAGE loading buffer, boiled, and centrifuged. Supernatants were resolved by SDS-PAGE and transferred to PVDF membranes. Immunoblotting was performed using the appropriate antibodies. Each assay was performed at least 3 times. All primers were listed in Table S14.

### Electrophoretic mobility shift assay

Electrophoretic mobility shift assays were performed using a Chemiluminescent EMSA Kit (Beyotime Biotechnology, Shanghai, China). Recombinant GST-tagged *NbWRKY45* and *NbVQ1* proteins were expressed in *Escherichia coli* and purified according to previously reported methods (Du et al. 2024a). Biotin-labeled DNA probes were synthesized by Sangon Biotech (Shanghai, China). EMSA reactions were carried out following the manufacturer protocol and the procedure described by Du et al. (2023b). Each assay was performed in triplicate. A list of primers used for probe synthesis was provided in Table S14. Each assay was performed at least 3 times.

### Subcellular localization and BiFC

To determine subcellular localization, coding sequences of *NbVQ1*, *NbWRKY45*, and *NbCAT2* were fused to GFP or mCherry and transiently expressed in *N. benthamiana* leaves via *Agrobacterium*-mediated transformation. Empty vector constructs were used as controls. Fluorescence signals were visualized 48 h postinfiltration using a confocal laser scanning microscope (FV3000, Olympus, Tokyo, Japan).

For BiFC analysis, the coding sequence of *NbVQ1* was fused to the N-terminal fragment of yellow fluorescent protein (YFP), and *NbWRKY45* was fused to the C-terminal fragment of YFP. The 2 constructs were coinfiltrated into *N. benthamiana* leaves. Reconstituted YFP fluorescence indicating protein–protein interaction was observed using confocal microscopy 48 h after infiltration. Each assay was performed at least 3 times. All primers were listed in Table S14.

### Weighted gene coexpression network analysis

WGCNA was performed using the WGCNA package in R platform to identify genes coexpressed with *NbVQs* and *NbWRKY45* based on previously obtained RNA-seq data (Langfelder and Horvath 2008; Du et al. 2023b). Network construction and module detection followed the package guidelines. The resulting topological overlap matrix was used to construct and visualize the gene network using Cytoscape software (v3.9.1).

### Chromatin immunoprecipitation followed by qPCR

Chromatin immunoprecipitation was conducted using the EpiQuik Plant ChIP Kit (Epigentek) following the manufacturer's instructions. Briefly, 2 to 4 g of leaf tissue from *NbWRKY45*-GFP transgenic plants was harvested and used for chromatin isolation. Chromatin was fragmented to 200 to 1,000 bp by sonication. Immunoprecipitation was performed using a ChIP-grade anti-GFP antibody (TransGen Biotech). After reverse cross-linking and proteinase K treatment, DNA was purified and subjected to qPCR using promoter-specific primers for *NbWRKY45* and *NbCAT2*. The enrichment of target sequences was normalized to the β-Actin promoter and compared with wild-type control samples. ChIP-qPCR assay was performed using 3 independent biological replicates. All primers were listed in Table S14.

### Drought treatment and stomatal ROS detection in *N. benthamiana*

Drought stress was simulated by irrigating plants with half-strength Hoagland nutrient solution containing 15% (w/v) PEG6000. Leaf samples were collected for ROS staining 1 d after the onset of drought treatment. Control plants were irrigated with nutrient solution without PEG6000 and sampled in parallel. For ROS staining, the abaxial epidermis was carefully peeled from fully expanded leaves and immediately immersed in 100 mm phosphate-buffered saline (PBS, pH 7.4) containing 10 *μ*m 2′,7′-dichlorodihydrofluorescein diacetate (Aladdin, CAS 4091-99-0). Samples were incubated in the dark at 25 °C for 30 min, rinsed 3 times with fresh PBS (pH 7.4), and mounted onto glass slides. ROS-associated fluorescence was visualized using a confocal laser scanning microscope (Nikon AX) with excitation at 488 nm, and emission was collected through a standard fluorescein isothiocyanate (FITC) filter set (515 to 530 nm). Each treatment comprised at least 10 independent biological replicates.

### Determination of antioxidant capacity of plant extracts

The ABTS radical-scavenging capacity was assessed according to Du et al. (2024a). Briefly, the absorbance of the reaction mixture was recorded at 734 nm, with vitamin C serving as a positive control. The antioxidant capacity of each sample was expressed as the half-maximal inhibitory concentration (IC_50_), defined as the extract concentration required to neutralize 50% of ABTS radicals. All measurements were performed in 3 independent biological replicates.

### Quantitative real-time PCR

Total RNA was extracted using the Plant Total RNA Kit (B518631, Sangon Biotech) and treated with RNase-free DNase. Two micrograms of RNA was reverse transcribed using the MightyScript First Strand cDNA Synthesis Master Mix (B639251, Sangon) in a 20-*μ*L reaction volume. The resulting cDNA was diluted fivefold and used as a template for RT-qPCR. Reactions (10 *μ*L volume) were conducted using ROX (carboxy-X-rhodamine) reference dye (B541010, Sangon) on a StepOnePlus Real-Time PCR System (Applied Biosystems), according to the manufacturer’s instructions. *NbACT* was used as an internal reference gene. qPCR cycling conditions were as follows: 95 °C for 30 s (initial denaturation), followed by 40 cycles at 95 °C for 5 s, 55 °C for 15 s, and 72 °C for 10 s. A melting curve was generated to confirm primer specificity. The relative expression gene levels were calculated using the 2^−ΔΔCT^ method. There were at least 3 biological replicates per treatment. Primer sequences were listed in Table S14.

### Metabolites extraction

Plant samples (20 ± 1 mg) were lyophilized and homogenized with beads in 1,000 *μ*L of extraction solution (MeOH:ACN:H_2_O, 2:2:1) containing deuterated internal standards. After vortexing for 30 s, the mixtures were subjected to 3 cycles of homogenization (35 Hz, 4 min) followed by sonication in a 4 °C water bath for 5 min. Subsequently, the samples were incubated at −40 °C for 1 h to precipitate proteins and then centrifuged at 12,000 rpm for 15 min at 4 °C. The quality control sample was prepared by combining equal volumes of supernatant from all samples.

### LC-MS/MS analysis

Nonpolar metabolites were analyzed using a UHPLC system (Vanquish, Thermo Fisher Scientific) equipped with a Phenomenex Kinetex C18 column (2.1 × 100 mm, 2.6 *μ*m) and coupled to an Orbitrap Exploris 120 mass spectrometer. The mobile phases consisted of A (0.01% acetic acid in water) and B (isopropanol:acetonitrile) (1:1). The column temperature was maintained at 25 °C, with an injection volume of 2 *μ*L. Mass spectrometry was performed in information-dependent acquisition mode with the following electrospray ionization (ESI) parameters: full MS and MS/MS resolutions of 60,000 and 15,000, respectively; stepped normalized collision energies (NCE) of 20, 30, and 40 eV; and spray voltages of +3.8 kV (positive) or −3.4 kV (negative).

### Data preprocessing and annotation

Raw data were converted to mzXML format using ProteoWizard and processed with an in-house R-based pipeline built on XCMS for feature detection, alignment, and integration. Metabolite identification was carried out using BiotreeDB (v3.0). Missing values were imputed as half of the minimum value. Statistical and functional analyses, including PCA, orthogonal partial least squares discriminant analysis, correlation analysis, and Kyoto Encyclopedia of Genes and Genomes (KEGG) enrichment, were performed in MetaboAnalyst. Metabolites with fold change >2.0 and *P* < 0.05 (Student's *t*-test) were considered significantly altered.

### Protein structure construction and interaction information analysis

The 3D structures of NbWRKY45, NbVQ1, and their interaction model were generated using AlphaFold 3.0. The interaction model and candidate residues involved in the NbWRKY45-NbVQ1 complex were visualized using PyMol software, and intermolecular interaction information was analyzed with Discovery Studio. The binding affinity between NbWRKY45 and NbVQ1 under different K^+^ conditions was calculated using FoldX according to Xu et al. (2025). Additionally, structural similarity between NbWRKY45, NbVQ1, NbCAT2, and their homologs was assessed using PyMol, with root mean square deviation values calculated using the protein alignment function in PyMol.

### Statistical analysis

All experiments were conducted with at least 3 independent biological replicates, each with a minimum of 3 technical replicates. Statistical significance was assessed using either 1-way ANOVA followed by Tukey's multiple comparison test or Student's *t*-test, as appropriate. Analyses were performed using GraphPad Prism 8.0.2. Data are presented as means ± Sd. Statistical significance was denoted as follows: *P  *<* 0.01; *P*  *<* 0.05; ns, not significant. There were at least 3 replicates per treatment, and the experiment was repeated 3 times.

## Supplementary Material

kiag469_Supplementary_Data

## Data Availability

All data in the study are public and have been submitted as required. Publicly available Supplemental Tables were analyzed in this study. All data are incorporated into the article and its online supplementary materials.

## References

[kiag469-B1] Amtmann A, Troufflard S, Armengaud P. 2008. The effect of potassium nutrition on pest and disease resistance in plants. Physiol Plantarum. 133:682–691. 10.1111/j.1399-3054.2008.01075.x. 18331404

[kiag469-B2] Apel K, Hirt H. 2004. Reactive oxygen species: metabolism, oxidative stress, and signal transduction. Annu Rev Plant Biol. 55:373–399. 10.1146/annurev.arplant.55.031903.141701. 15377225

[kiag469-B3] Chen J et al 2018. *Arabidopsis* VQ10 interacts with WRKY8 to modulate basal defense against *Botrytis cinerea*. J Integr Plant Biol. 60:956–969. 10.1111/jipb.12664. 29727045

[kiag469-B4] Cheng Y et al 2012. Structural and functional analysis of VQ motif-containing proteins in *Arabidopsis* as interacting proteins of WRKY transcription factors. Plant Physiol. 159:810–825. 10.1104/pp.112.196816. 22535423 PMC3375943

[kiag469-B5] Cheng YT, Zhang L, He SY. 2019. Plant-microbe interactions facing environmental challenge. Cell Host Microbe. 26:183–192. 10.1016/j.chom.2019.07.009. 31415751 PMC6697056

[kiag469-B6] Chouteau J, Fauconnier D. 1998. Fertilizing for high quality and yield tobacco. International Potash Institute.

[kiag469-B7] Demidchik V et al 2010. Arabidopsis root K^+^-efflux conductance activated by hydroxyl radicals: single-channel properties, genetic basis and involvement in stress-induced cell death. J Cell Sci. 123:1468–1479. 10.1242/jcs.064352. 20375061

[kiag469-B8] Demidchik V et al 2014. Stress-induced electrolyte leakage: the role of K^+^-permeable channels and involvement in programmed cell death and metabolic adjustment. J Exp Bot. 65:1259–1270. 10.1093/jxb/eru004. 24520019

[kiag469-B9] Demidchik V, Shabala SN, Coutts KB, Tester MA, Davies JM. 2003. Free oxygen radicals regulate plasma membrane Ca^2+^- and K^+^-permeable channels in plant root cells. J Cell Sci. 116:81–88. 10.1242/jcs.00201. 12456718

[kiag469-B10] Dodds PN, Rathjen JP. 2010. Plant immunity: towards an integrated view of plant-pathogen interactions. Nat Rev Genet. 11:539–548. 10.1038/nrg2812. 20585331

[kiag469-B11] Dong Q et al 2024. The MdVQ37-MdWRKY100 complex regulates salicylic acid content and *MdRPM1* expression to modulate resistance to Glomerella leaf spot in apples. Plant Biotechnol J. 22:2364–2376. 10.1111/pbi.14351. 38683692 PMC11258982

[kiag469-B12] Du P et al 2023a. WRKY transcription factors and OBERON histone-binding proteins form complexes to balance plant growth and stress tolerance. EMBO J. 42:e113639. 10.15252/embj.2023113639. 37565504 PMC10548177

[kiag469-B13] Du Y et al 2023b. Sufficient coumarin accumulation improves apple resistance to *Cytospora mali* under high-potassium status. Plant Physiol. 192:1396–1419. 10.1093/plphys/kiad184. 36943289 PMC10231470

[kiag469-B14] Du Y et al 2024a. Major quality regulation network of flavonoid synthesis governing the bioactivity of black wolfberry. New Phytol. 242:558–575. 10.1111/nph.19602. 38396374

[kiag469-B15] Du Y et al 2024b. Changes in planta K nutrient content altered the interaction pattern between *Nicotiana benthamiana* and *Alternaria longipes*. Plant Cell Environ. 47:3619–3637. 10.1111/pce.14956. 38747645

[kiag469-B16] Duan D et al 2025. The MdVQ17/MdVQ37-MdWRKY100 module coordinates apple salt tolerance by modulating Na^+^/K^+^ homeostasis and reactive oxygen species scavenging. Plant Physiol. 199:kiaf434. 10.1093/plphys/kiaf434. 41027008

[kiag469-B17] Feng C et al 2025. GmAKT1-mediated K^+^ absorption positively modulates soybean salt tolerance by GmCBL9-GmCIPK6 complex. Plant Biotechnol J. 23:2276–2289. 10.1111/pbi.70042. 40112140 PMC12120911

[kiag469-B18] Gao H et al 2022. Natural variations of ZmSRO1d modulate the trade-off between drought resistance and yield by affecting ZmRBOHC-mediated stomatal ROS production in maize. Mol Plant. 15:1558–1574. 10.1016/j.molp.2022.08.009. 36045577

[kiag469-B19] Hao Z et al 2022. A VQ-motif-containing protein fine-tunes rice immunity and growth by a hierarchical regulatory mechanism. Cell Rep. 40:111235. 10.1016/j.celrep.2022.111235. 35977497

[kiag469-B20] Huang H et al 2025. SlVQ15 recruits SlWRKY30IIc to link with jasmonate pathway in regulating tomato defence against root-knot nematodes. Plant Biotechnol J. 23:235–249. 10.1111/pbi.14493. 39501496 PMC11672745

[kiag469-B21] Javed T, Gao SJ. 2023. WRKY transcription factors in plant defense. Trends Genet. 39:787–801. 10.1016/j.tig.2023.07.001. 37633768

[kiag469-B22] Jia H et al 2025. Transcriptional activation of *MdDEF30* by MdWRKY75 enhances apple resistance to Cytospora canker. J Integr Plant Biol. 24:1108–1125. 10.1016/j.jia.2024.06.001.

[kiag469-B23] Jiang J et al 2017. WRKY transcription factors in plant responses to stresses. J Integr Plant Biol. 59:86–101. 10.1111/jipb.12513. 27995748

[kiag469-B24] Jones J, Staskawicz B, Dangl J. 2024. The plant immune system: from discovery to deployment. Cell. 187:2095–2116. 10.1016/j.cell.2024.03.045. 38670067

[kiag469-B25] Khokon AR et al 2011. Involvement of extracellular oxidative burst in salicylic acid-induced stomatal closure in *Arabidopsis*. Plant Cell Environ. 34:434–443. 10.1111/j.1365-3040.2010.02253.x. 21062318

[kiag469-B26] Langfelder P, Horvath S. 2008. WGCNA: an R package for weighted correlation network analysis. BMC Bioinf. 9:559. 10.1186/1471-2105-9-559. PMC2631488 19114008

[kiag469-B27] Li J et al 2023. Phosphatidic acid-regulated SOS2 controls sodium and potassium homeostasis in *Arabidopsis* under salt stress. EMBO J. 42:e112401. 10.15252/embj.2022112401. 36811145 PMC10106984

[kiag469-B28] Lim C, Kang K, Shim Y, Yoo SC, Paek NC. 2022. Inactivating transcription factor OsWRKY5 enhances drought tolerance through abscisic acid signaling pathways. Plant Physiol. 188:1900–1916. 10.1093/plphys/kiab492. 34718775 PMC8968288

[kiag469-B29] Liu C, Liao W. 2022. Potassium signaling in plant abiotic responses: crosstalk with calcium and reactive oxygen species/reactive nitrogen species. Plant Physiol Bioch. 173:110–121. 10.1016/j.plaphy.2022.01.016. 35123248

[kiag469-B30] Liu D et al 2021. Tobacco transcription factor bHLH123 improves salt tolerance by activating NADPH oxidase *NtRbohE* expression. Plant Physiol. 186:1706–1720. 10.1093/plphys/kiab176. 33871656 PMC8260122

[kiag469-B31] Locascio A et al 2019. BCL2-ASSOCIATED ATHANOGENE4 regulates the KAT1 potassium channel and controls stomatal movement. Plant Physiol. 181:1277–1294. 10.1104/pp.19.00224. 31451552 PMC6836829

[kiag469-B32] Luan S, Wang C. 2021. Calcium signaling mechanisms across kingdoms. Annu Rev Cell Dev Biol. 37:311–340. 10.1146/annurev-cellbio-120219-035210. 34375534

[kiag469-B33] Luo Q, Feng J, Yang G, He G. 2022. Functional characterization of BdCIPK31 in plant response to potassium deficiency stress. Plant Physiol Bioch. 192:243–251. 10.1016/j.plaphy.2022.10.014. 36272191

[kiag469-B34] Ma J et al 2023. The SlWRKY57-SlVQ21/SlVQ16 module regulates salt stress in tomato. J Integr Plant Biol. 65:2437–2455. 10.1111/jipb.13562. 37665103

[kiag469-B35] Mittler R . 2017. ROS are good. Trends Plant Sci. 22:11–19. 10.1016/j.tplants.2016.08.002. 27666517

[kiag469-B36] Mittler R, Zandalinas SI, Fichman Y, Van Breusegem F. 2022. Reactive oxygen species signalling in plant stress responses. Nat Rev Mol Cell Biol. 23:663–679. 10.1038/s41580-022-00499-2. 35760900

[kiag469-B37] Mostofa MG et al 2022. Potassium in plant physiological adaptation to abiotic stresses. Plant Physiol Bioch. 186:279–289. 10.1016/j.plaphy.2022.07.011. 35932652

[kiag469-B38] Ngou B, Ahn HK, Ding P, Jones J. 2021. Mutual potentiation of plant immunity by cell-surface and intracellular receptors. Nature. 592:110–115. 10.1038/s41586-021-03315-7. 33692545

[kiag469-B39] Ngou B, Ding P, Jones J. 2022a. Thirty years of resistance: zig-zag through the plant immune system. Plant Cell. 34:1447–1478. 10.1093/plcell/koac041. 35167697 PMC9048904

[kiag469-B40] Ngou B, Jones J, Ding P. 2022b. Plant immune networks. Trends Plant Sci. 27:255–273. 10.1016/j.tplants.2021.08.012. 34548213

[kiag469-B41] Niu F et al 2020. WRKY42 transcription factor positively regulates leaf senescence through modulating SA and ROS synthesis in *Arabidopsis thaliana*. Plant J. 104:171–184. 10.1111/tpj.14914. 32634860

[kiag469-B42] Osakabe Y et al 2013. Osmotic stress responses and plant growth controlled by potassium transporters in *Arabidopsis*. Plant Cell. 25:609–624. 10.1105/tpc.112.105700. 23396830 PMC3608781

[kiag469-B43] Peng H et al 2016. Management of Valsa canker on apple with adjustments to potassium nutrition. Plant Dis. 100:884–889. 10.1094/PDIS-09-15-0970-RE. 30686143

[kiag469-B44] Qi J et al 2018. Reactive oxygen species signaling and stomatal movement in plant responses to drought stress and pathogen attack. J Integr Plant Biol. 60:805–826. 10.1111/jipb.12654. 29660240

[kiag469-B45] Qi J, Wang J, Gong Z, Zhou JM. 2017. Apoplastic ROS signaling in plant immunity. Curr Opin Plant Biol. 38:92–100. 10.1016/j.pbi.2017.04.022. 28511115

[kiag469-B46] Ravi B, Foyer C, Pandey GK. 2023. The integration of reactive oxygen species (ROS) and calcium signalling in abiotic stress responses. Plant Cell Environ. 46:1985–2006. 10.1111/pce.14596. 37132157

[kiag469-B47] Shabala S . 2017. Signalling by potassium: another second messenger to add to the list? J Exp Bot. 68:4003–4007. 10.1093/jxb/erx238. 28922770 PMC5853517

[kiag469-B48] Shabala S, Pottosin I. 2014. Regulation of potassium transport in plants under hostile conditions: implications for abiotic and biotic stress tolerance. Physiol Plant. 151:257–279. 10.1111/ppl.12165. 24506225

[kiag469-B49] Shi X et al 2018. The fungal pathogen *Magnaporthe oryzae* suppresses innate immunity by modulating a host potassium channel. PLoS Pathog. 14:e1006878. 10.1371/journal.ppat.1006878. 29385213 PMC5809103

[kiag469-B50] Sierla M, Waszczak C, Vahisalu T, Kangasjärvi J. 2016. Reactive oxygen species in the regulation of stomatal movements. Plant Physiol. 171:1569–1580. 10.1104/pp.16.00328. 27208297 PMC4936562

[kiag469-B51] Van Breusegem F, Dat JF. 2006. Reactive oxygen species in plant cell death. Plant Physiol. 141:384–390. 10.1104/pp.106.078295. 16760492 PMC1475453

[kiag469-B52] Wang N et al 2022. Transcriptional repression of *TaNOX10* by TaWRKY19 compromises ROS generation and enhances wheat susceptibility to stripe rust. Plant Cell. 34:1784–1803. 10.1093/plcell/koac001. 34999846 PMC9048928

[kiag469-B53] Wang P et al 2024a. Reactive oxygen species: multidimensional regulators of plant adaptation to abiotic stress and development. J Integr Plant Biol. 66:330–367. 10.1111/jipb.13601. 38116735

[kiag469-B54] Wang R, He F, Ning Y, Wang GL. 2020. Fine-tuning of RBOH-mediated ROS signaling in plant immunity. Trends Plant Sci. 25:1060–1062. 10.1016/j.tplants.2020.08.001. 32861572

[kiag469-B55] Wang R, Li J, Liang Y. 2024b. Role of ROS signaling in the plant defense against vascular pathogens. Curr Opin in Plant Biol. 81:102617. 10.1016/j.pbi.2024.102617. 39163783

[kiag469-B56] Wang Y, Wu WH. 2010. Plant sensing and signaling in response to K^+^-deficiency. Mol Plant. 3:280–287. 10.1093/mp/ssq006. 20339156

[kiag469-B57] Waszczak C, Carmody M, Kangasjärvi J. 2018. Reactive oxygen species in plant signaling. Annu Rev Plant Biol. 69:209–236. 10.1146/annurev-arplant-042817-040322. 29489394

[kiag469-B58] Willekens H et al 1997. Catalase is a sink for H_2_O_2_ and is indispensable for stress defence in C3 plants. EMBO J. 16:4806–4816. 10.1093/emboj/16.16.4806. 9305623 PMC1170116

[kiag469-B59] Wong HL et al 2007. Regulation of rice NADPH oxidase by binding of Rac GTPase to its N-terminal extension. Plant Cell. 19:4022–4034. 10.1105/tpc.107.055624. 18156215 PMC2217649

[kiag469-B60] Wu B, Qi F, Liang Y. 2023. Fuels for ROS signaling in plant immunity. Trends Plant Sci. 28:1124–1131. 10.1016/j.tplants.2023.04.007. 37188557

[kiag469-B61] Xia J et al 2021. Whitefly hijacks a plant detoxification gene that neutralizes plant toxins. Cell. 184:1693–1705.e17. 10.1016/j.cell.2021.02.014. 33770502

[kiag469-B62] Xie YD et al 2010. The *Arabidopsis* gene SIGMA FACTOR-BINDING PROTEIN 1 plays a role in the salicylate- and jasmonate-mediated defence responses. Plant Cell Environ. 33:828–839. 10.1111/j.1365-3040.2009.02109.x. 20040062 PMC3208021

[kiag469-B63] Xu P et al 2025. The OsbZIP35-COR1-OsTCP19 module modulates cell proliferation to regulate grain length and weight in rice. Sci Adv. 11:eadx9815. 10.1126/sciadv.adx9815. 40929276 PMC12422201

[kiag469-B64] Yang L et al 2025. WRKY transcription factors: hubs for regulating plant growth and stress responses. J Integr Plant Biol. 67:488–509. 10.1111/jipb.13828. 39815727

[kiag469-B65] Yang Y et al 2024. The microRNA408-plantacyanin module balances plant growth and drought resistance by regulating reactive oxygen species homeostasis in guard cells. Plant Cell. 36:4338–4355. 10.1093/plcell/koae144. 38723161 PMC11448907

[kiag469-B66] Yuan M et al 2021. Pattern-recognition receptors are required for NLR-mediated plant immunity. Nature. 592:105–109. 10.1038/s41586-021-03316-6. 33692546 PMC8016741

[kiag469-B67] Zhou J et al 2020. Differential phosphorylation of the transcription factor WRKY33 by the protein kinases CPK5/CPK6 and MPK3/MPK6 cooperatively regulates camalexin biosynthesis in *Arabidopsis*. Plant Cell. 32:2621–2638. 10.1105/tpc.19.00971. 32439826 PMC7401014

[kiag469-B68] Zhu L et al 2024. Applied potassium negates osmotic stress impacts on plant physiological processes: a meta-analysis. Hortic Res. 12: uhae318. 10.1093/hr/uhae318. 39949879 PMC11825146

