# Supplementary material for: NbVQ1 physically turns off the NbWRKY45-NbCAT2 module to promote reactive oxygen species burst and disease resistance in plants under high-potassium regime
Source: Plant Physiol. 2026 Jul 1;201(3):kiag469. doi: 10.1093/plphys/kiag469 (PMC13418371; doi:10.1093/plphys/kiag469)
Supplement: kiag469_Supplementary_Data [file kiag469_supplementary_data.zip › Revised Supplemental Figures20260531.docx]

**NbVQ1 physically turns off the NbWRKY45-NbCAT2 module to promote ROS burst and disease resistance in plants under high-potassium regime**

Youwei Du^1^#, Shuanghong Wang^1^#, Guangli Liu^1^#, Jinchao Zhou^1^, Zhonghong Feng^1^, Zheyan Qiao^1^, Shuang Zhang^1^, Rong Zhang^1^, Mark L. Gleason^4^, Qiang Yao^3^*, Hongchen Jia^1, 2^* and Guangyu Sun^1^*

(1 College of Plant Protection and State Key Laboratory of crop Stress Resistance and high-efficiency Production, Northwest A&F University, Yangling, Shaanxi, 712100, China; 2 State Key Laboratory of Tropical Crop Breeding, Institute of Tropical Bioscience and Biotechnology & Sanya Research Institute, Chinese Academy of Tropical Agricultural Sciences, Sanya 572024, China; 3 Qinghai Academy of Agriculture and Forestry Science, Qinghai University, Xining, Qinghai, 810016, China; 4 Department of Plant Pathology, Entomology, and Microbiology, Iowa State University, Ames, Iowa 50011, USA)

#These authors contributed equally to this work.

**Running title:** Potassium triggered NbVQ1-NbWRKY45 module to enhance ROS immunity

**One sentence summary:** NbVQ1 suppresses the NbWRKY45-NbCAT2 module to promote ROS burst and immune responses for enhancing disease resistance of plants with high-potassium status.

**Corresponding author:** Guangyu Sun; Hongchen Jia; Qiang Yao

**E-mail:** [sgy@nwsuaf.edu.cn](mailto:sgy@nwsuaf.edu.cn); [jhc@nwafu.edu.cn](mailto:jhc@nwafu.edu.cn); [yaoqiang2010@126.com](mailto:yaoqiang2010@126.com);

**Key words:** Potassium nutrient; Basal resistance; ROS scavenging; Multi-omics; Transcription cascade

**Supplemental Figures:**


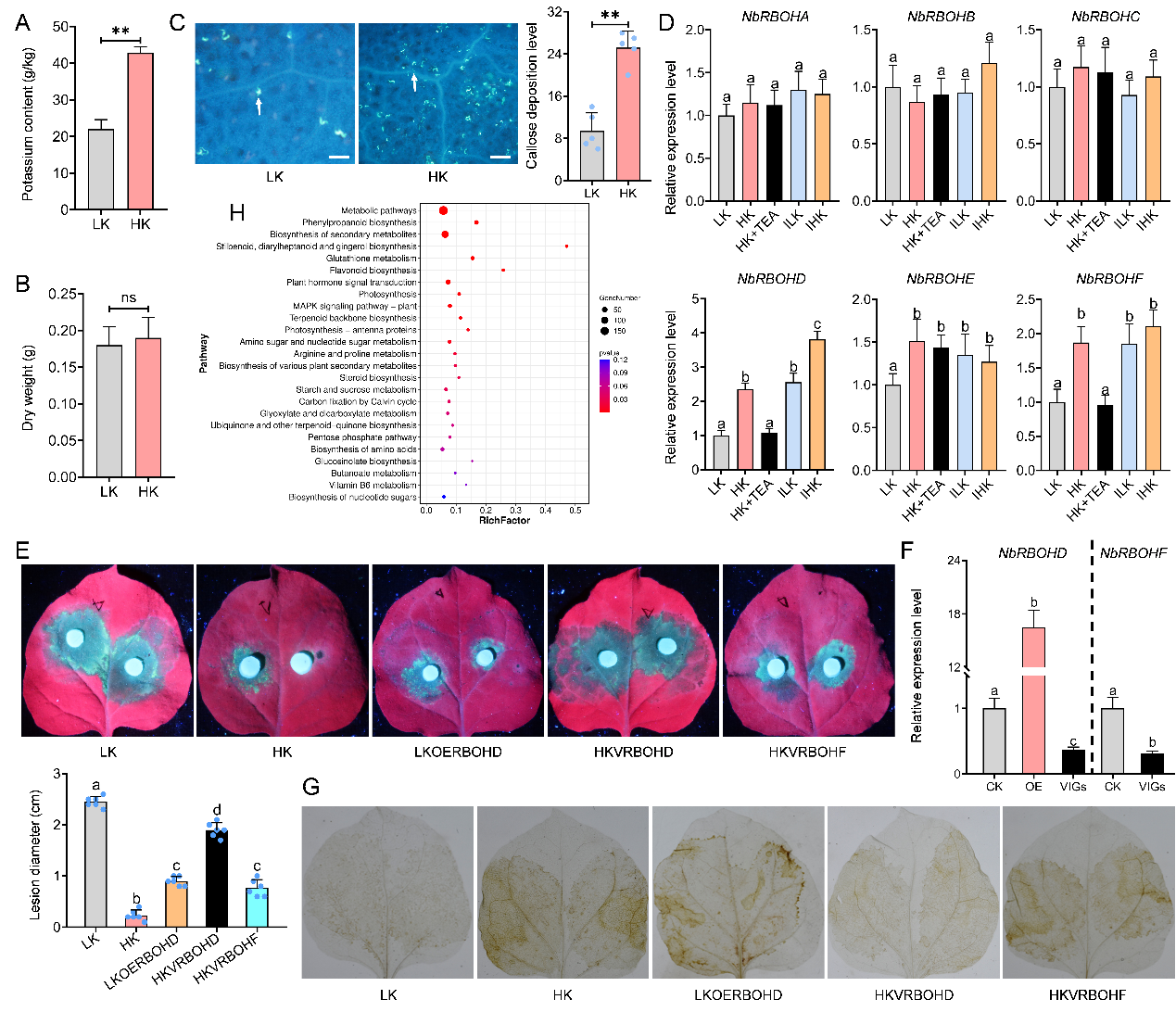


**Figure S1 Supplementing potassium promotes the pathogen-triggered activation of basal resistance and NbRBOHD-mediated ROS burst in *N. benthamiana*. A**: Plant potassium content of high-K (HK) and low-K (LK) *N. benthamiana* plants; n = 5. **B:** The dry weight of *N. benthamiana* plants under HK and LK conditions; n = 3. **C:** The level of *Phytophthora parasitica*-induced callose deposition in HK and LK *N. benthamiana*; The callose deposits are labeled using white arrow; Bar = 50 μm; n = 5. The aniline blue staining shows higher level of callose deposition in HK *N. benthamiana*, compared to LK *N. benthamiana* following *P. parasitica* challenge. **D:** RT-qPCR validation of expression levels of *NbRBOHD* and *NbRBOHF*, *NbRBOHA*, *NbRBOHB*, and *NbRBOHC* in infected and non-infected *N. benthamiana* under HK and LK conditions; *NbRBOHD* and *NbRBOHF* were upregulated in HK *N. benthamiana* and further induced upon pathogen challenge. However, TEA (tetraethylammonium) treatment effectively suppressed their upregulation even under high‑K conditions. In contrast, *NbRBOHA*, *NbRBOHB*, and *NbRBOHC* showed no responsiveness to either pathogen infection or changes in K nutrient; n = 3. **E:** Function analysis of NbRBOHD and NbRBOHF in mediating the enhanced resistance of *N. benthamiana* under HK conditions. *NbRBOHD* or *NbRBOHF* overexpression increased resistance in LK *N. benthamiana* to *P. parasitica*, and NbRBOHD exerted more significant effects than NbRBOHF; Silencing of *NbRBOHD* caused decreases in disease resistance in HK *N. benthamiana*; n = 6. **F:** RT-qPCR confirmation about overexpression or silencing efficiency of *NbRBOHD* or *NbRBOHF* in representative *N. benthamiana* leaves; n = 3. **G:** DAB staining observation of *P. parasitica*-induced reactive oxygen species (ROS, H_2_O_2_) in *N. benthamiana* with *NbRBOHD* silencing or overexpression under different K conditions. NbRBOHD silencing recued the *P. parasitica*-triggered ROS production level in HK *N. benthamiana*, while its overexpression promoted ROS burst in LK *N. benthamiana*. **H:** Pathway enrichment analysis on differentially expressed genes (DEGs) that significantly upregulated in HK *N. benthamiana*. The various color levels displayed different levels of significance of KEGG pathway from low (red) to high (purple). Data in **Fig. S1** are presented as the mean ± SD; Different letters and “**” represent significant differences based on *P* < 0.05; “ns” indicates no significance; Statistical analysis in **Fig. S1D-F** is determined by one-way ANOVA followed by post-hoc Tukey test, and Student’s *t*-test in **Fig. S1A-C**.


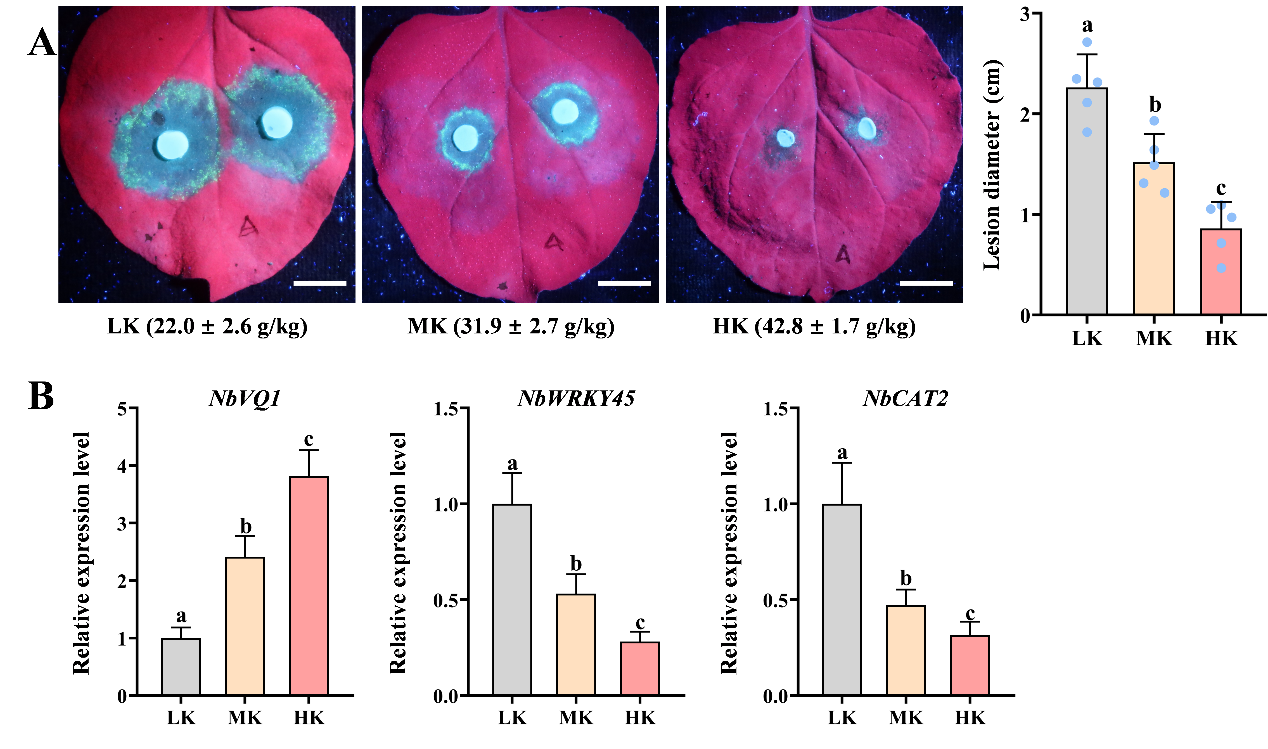


**Figure S2 Adding K nutrient exerts a dose-dependent effects in promoting *N. benthamiana* resistance and affecting NbVQ1-NbWRKY45-NbCAT2 module. A:** Resistance analysis of *N. benthamiana* with high- (HK), medium- (MK) and low-K (LK) contents to *P. parasitica* infection. Adding in planta K contents increases disease resistance of *N. benthamiana* in a dose-dependency. n = 5. **B:** Expression levels of *NbVQ1*, *NbWRKY45* and *NbCAT2* in HK, MK and LK *N. benthamiana*. The effects of changes in planta K contents on altering *NbVQ1*, *NbWRKY45* and *NbCAT2* expressions exerts a dose-dependent characteristic; n = 3. Data in **Fig. S2** are presented as the mean ± SD; Different letters represent significant differences at *P* < 0.05 based on one-way ANOVA followed by post-hoc Tukey test.


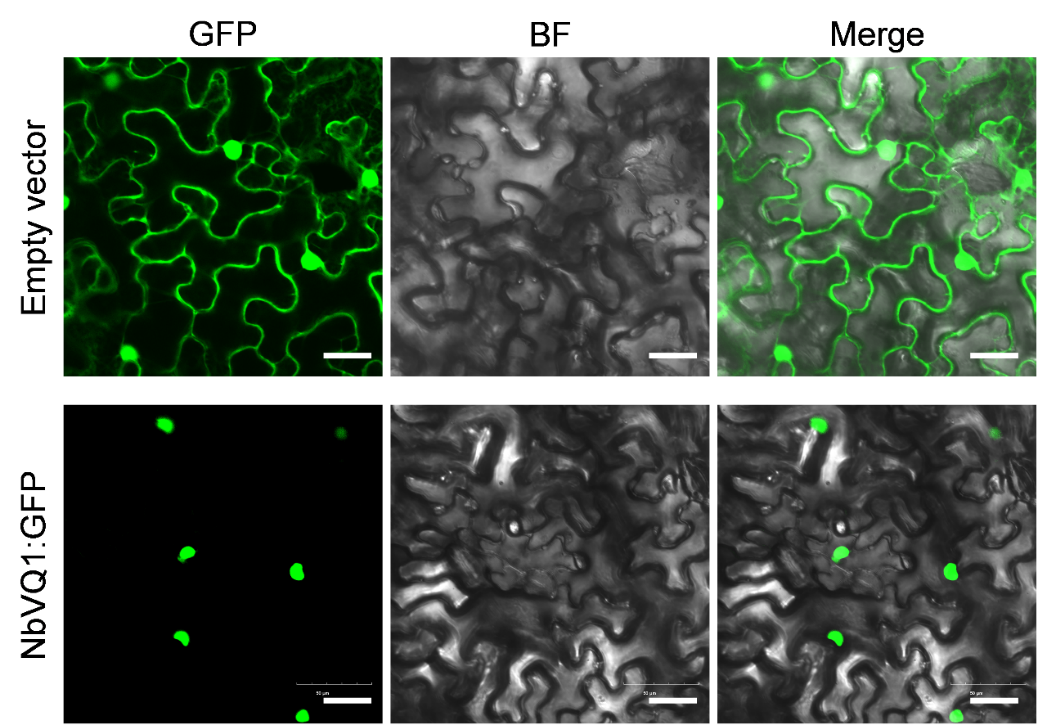


**Figure S3 Subcellular localization of NbVQ1 in *N. benthamiana*.** *Agrobacterium tumefaciens* carrying NbVQ1-GFP fusion protein was infiltrated into *N. benthamiana* leaves for transient overexpression and subcellular location observation. *A*. *tumefaciens* carrying green fluorenscent protein (GFP) expression was infiltrated into tobacco leaves as controls. The images were visualized by laser scanning confocal microscopy; Bar = 20 μm.


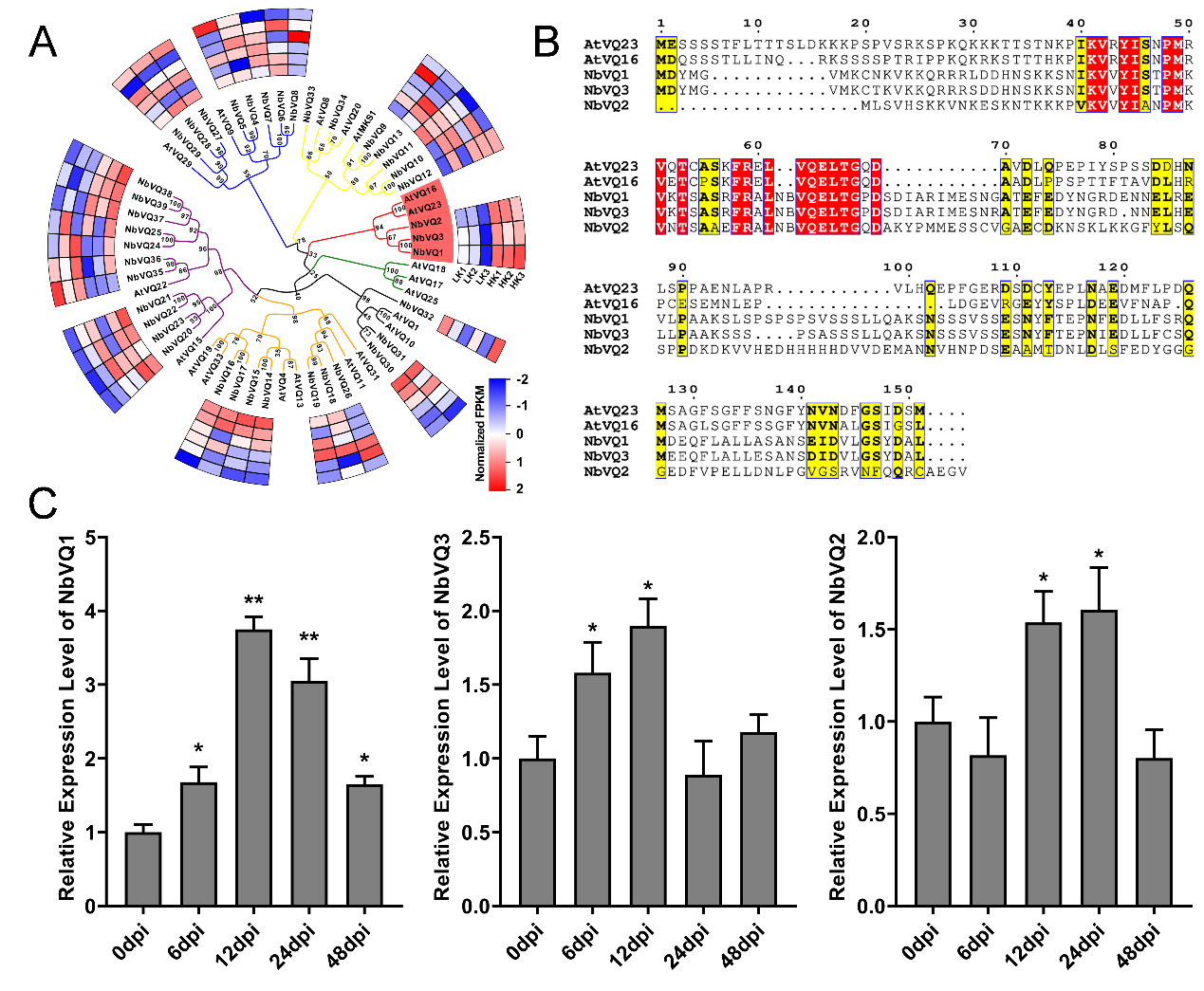


**Figure S4 Phylogenetic and expression analysis of NbVQ1 and homologous proteins in *N. benthamiana* following *P. parasitica* infection. A:** The phylogenetic analysis of NbVQ proteins obtained from *N. benthamiana* and *Arabidopsis thaliana*. The heat map represents the expression patterns of NbVQs in HK and LK *N. benthamiana*. The scale bar represents the normalized FPKM values. Phylogenetic tree is constructed using Randomized Axelerated Maximum Likelihood (RAxML) method based on bootstraps = 1000. **B:** Comparative analysis of amino acid sequence between NbVQ1 and homologous protein. **C:** RT-qPCR determines the expression levels of *NbVQ1*, *NbVQ2* and *NbVQ3* in different time of *P. parasitica* infection; n = 3. Data in **Fig. S4** are presented as the mean ± SD; Statistical analysis is determined using one-way ANOVA followed by post-hoc Tukey test.


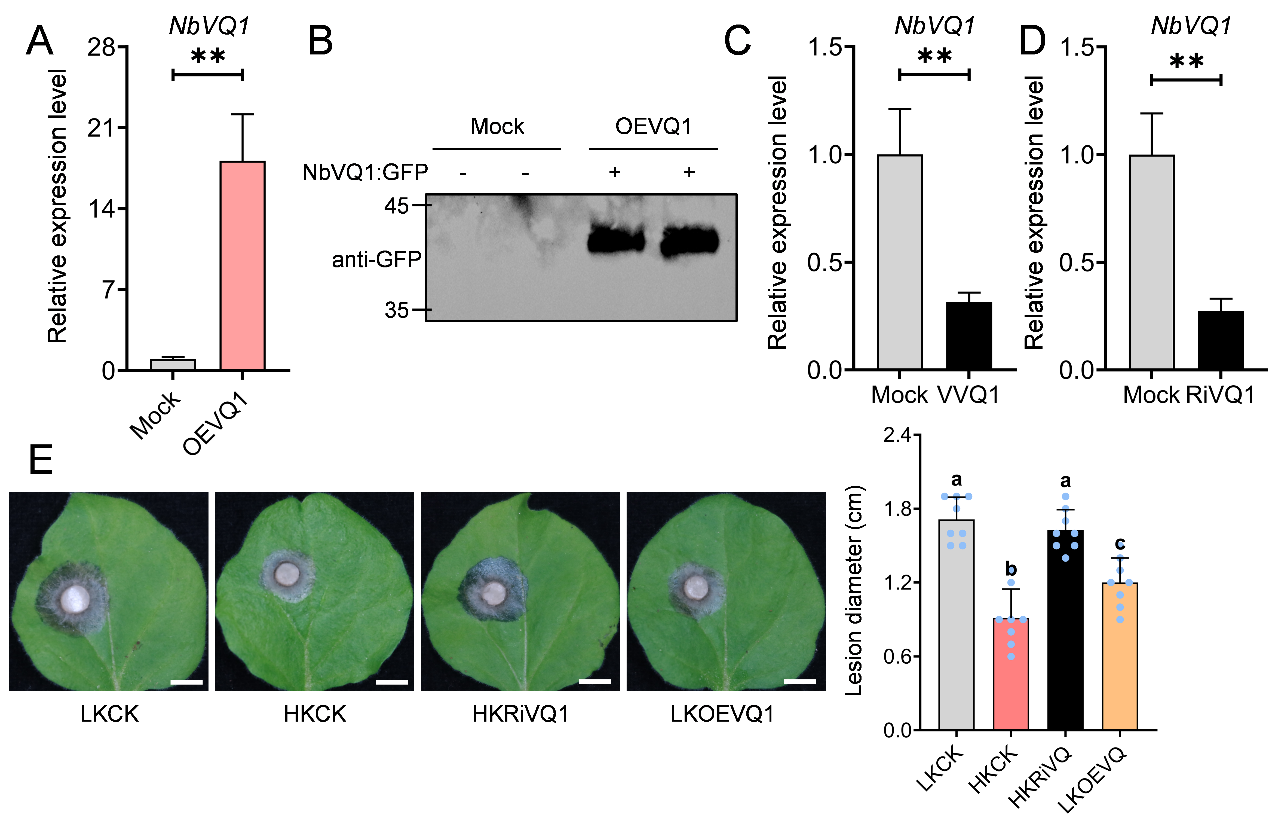


**Figure S5** **Verification and function of stable *NbVQ1* overexpression or silencing in transgenic *N. benthamiana* seedlings.** **A:** RT-qPCR assay determines the *NbVQ1* overexpression efficiency in *N. benthamiana*; n = 3. **B:** Westen blot assay confirms the overexpression of NbVQ1 protein in representative transgenic *N. benthamiana* seedlings. **C:** RT-qPCR confirms the efficiency of VIGs-induced *NbVQ1* silencing in *N. benthamiana* seedlings; n = 3. **D:** RT-qPCR confirms the efficiency of RNAi-mediated stable *NbVQ1* silencing in transgenic *N. benthamiana* seedlings. **E:** NbVQ1 positively regulates HK-associated resistance in *N. benthamiana* to *Botrytis cinerea*. *NbVQ1* overexpression promotes resistance in LK *N. benthamiana* to *B*. *cinerea*, while *NbVQ1* silencing results in decreases in resistance in HK *N. benthamiana*. Data in **Fig. S5** are presented as the mean ± SD; Different letters and “**” represent significant differences based on *P* < 0.05; Statistical analysis in **Fig. S5A-D** is determined by Student’s *t*-test, and one-way ANOVA followed by post-hoc Tukey test in **Fig. S5E**.


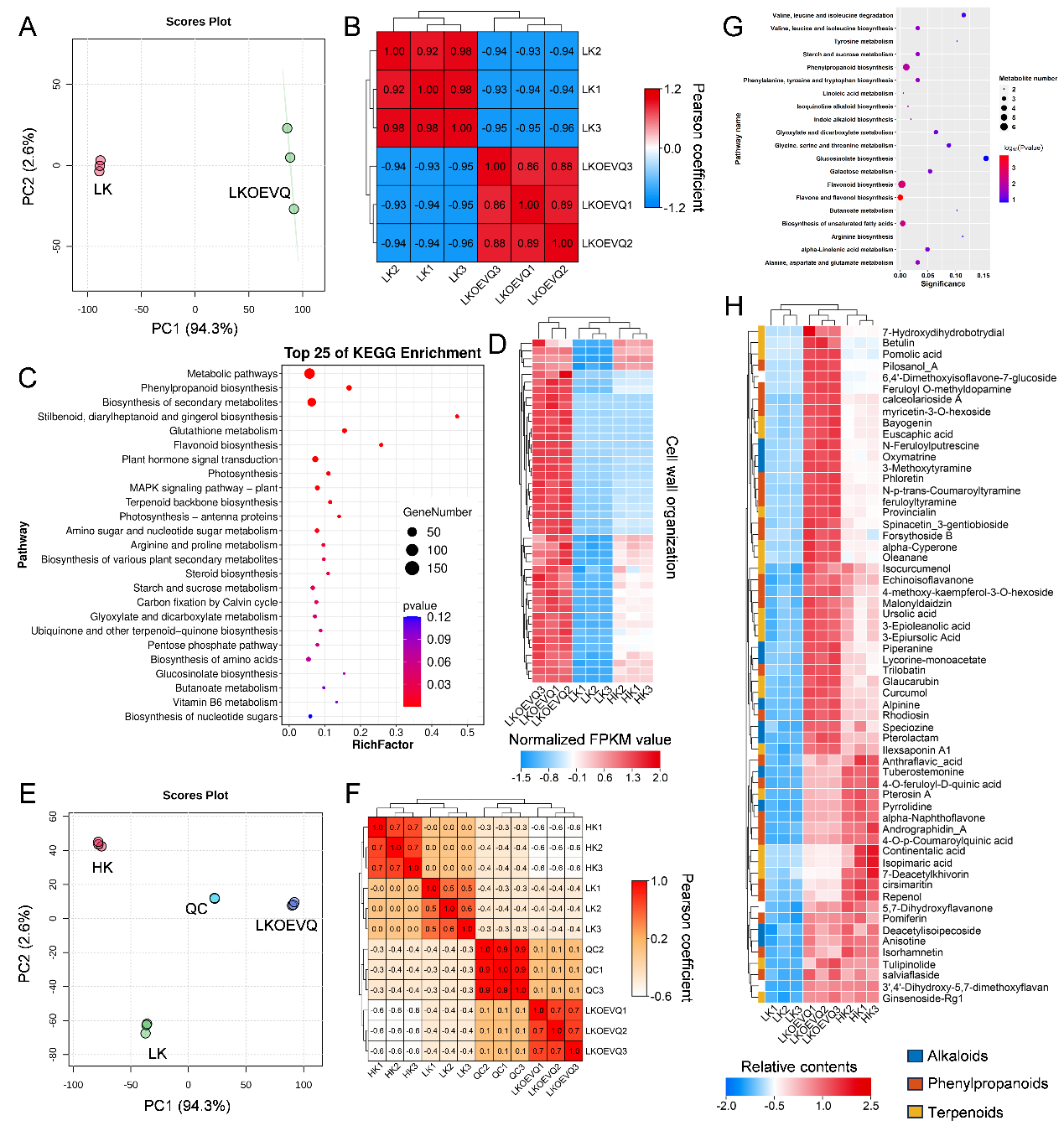


**Figure S6 NbVQ1 promotes transcription and metabolic changes associated with basal resistance in HK *N. benthamiana*. A:** Principal component analysis (PCA) plots of transcriptome profiles of LK *N. benthamiana* and LK *N. benthamiana* overexpressing *NbVQ1* (LKOEVQ). Ellipses show 95% confidence intervals. The samples with different treatments were shown in different colors. The PCA plots are constructed using top 2 principal components (PC1 and PC2). **B:** Pearson correlation analysis on all transcriptome profiles of LKOEVQ and LK *N. benthamiana*. The high Pearson value of correlation is shown in red. Scale bar represents the Pearson correlation value between two experimental samples. **C:** KEGG pathway enrichment analysis of genes upregulated in LK *N. benthamiana* triggered by *NbVQ1* overexpression. **D:** Heat maps display the relative expression abundances of genes relevant to cell wall organization in LKOEVQ and LK *N. benthamiana*. The high and low expression abundances are shown in red and blue, respectively. The scale represents the normalized FPKM values of genes in each biological replicates. **E:** PCA displaying the distinct metabolic pattern in LKOEVQ, HK and LK *N. benthamiana*. QC: quality control samples. **F:** Pearson correlation analysis on all metabolic data of LKOEVQ, HK and LK *N. benthamiana*. The high Pearson value of correlation is shown in red. Scale bar represents the Pearson correlation value between two experimental samples. **G:** Scatter plot displays the KEGG pathway enrichment results about upregulated DEGs from HK vs. LK pairwise comparisons. **H:** Heat map about relative content of compounds relevant to defensive metabolites (phenylpropanoids, alkaloids and terpenoids) in LKOEVQ, HK and LK *N. benthamiana*. The higher and lower contents are shown in red and blue, respectively. The scale represents the normalized peak area values of metabolites in each biological replicates.


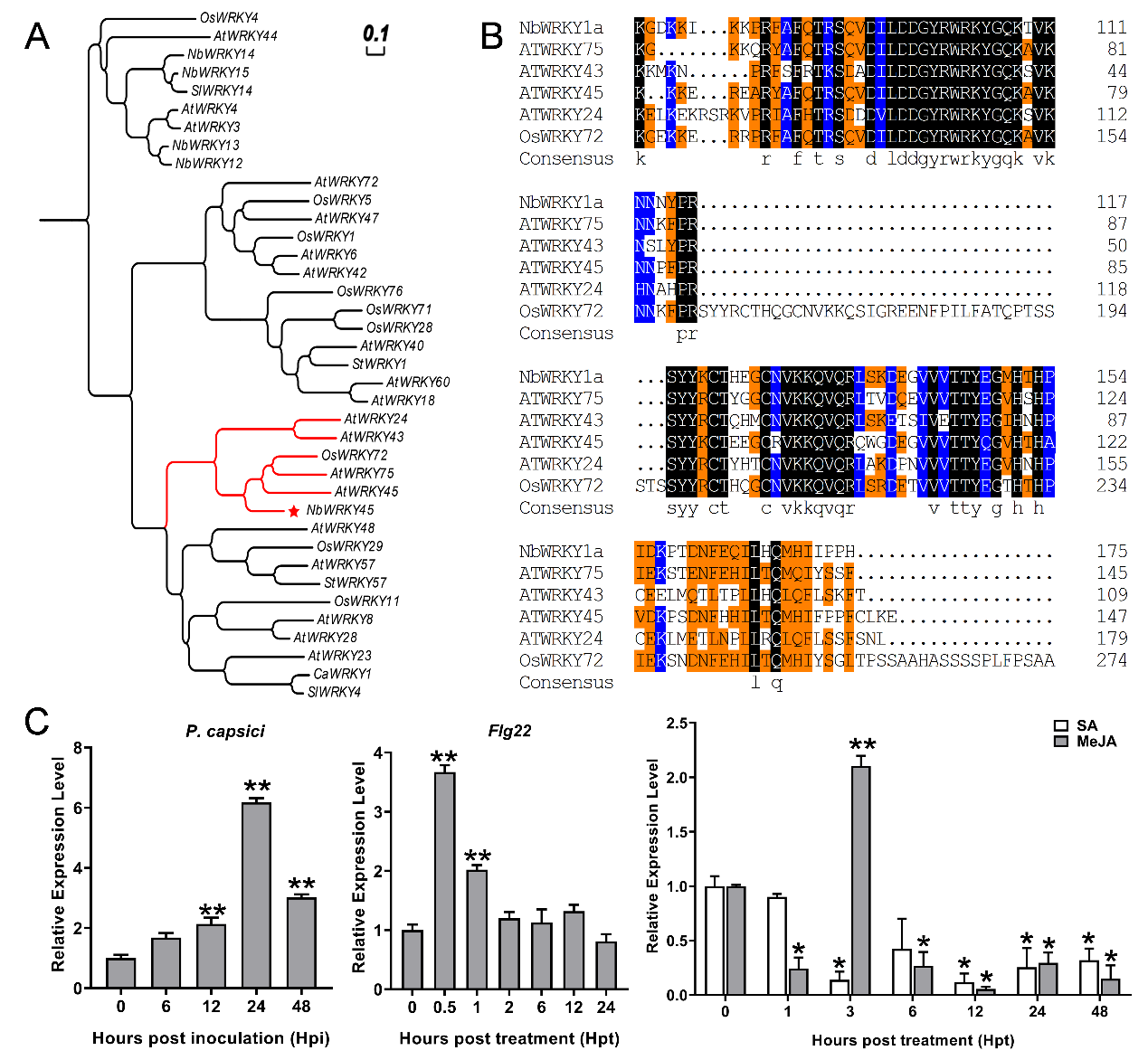


**Figure S7 Phylogenetic and expression analysis of NbWRKY45 and homologous proteins in *N. benthamiana* with different treatments. A:** The phylogenetic analysis of WRKY proteins obtained from *N. benthamiana*, *Oryza sativa*, *Solanum lycopersicum*, *Solanum tuberosum* and *A*. *thaliana*. Phylogenetic tree is constructed using RAxML algorithm based on bootstraps = 1000. **B:** Comparative sequence analysis of amino acid sequence between NbWRKY45 and homologous protein. Alignment results shows that these WRKY proteins shared intact WRKYGQK core sequence and C2H2 motif (C-X5-C-X23-H-X1-H). **C:** RT-qPCR assay detects the expression pattern of *NbWRKY45* in *N. benthamiana* after inoculation with *P. parasitica*, induction by flg22 and hormone at different time points. n = 3. Data in **Fig. S7** are presented as the mean ± SD; Statistical analysis is determined using one-way ANOVA followed by post-hoc Tukey test.


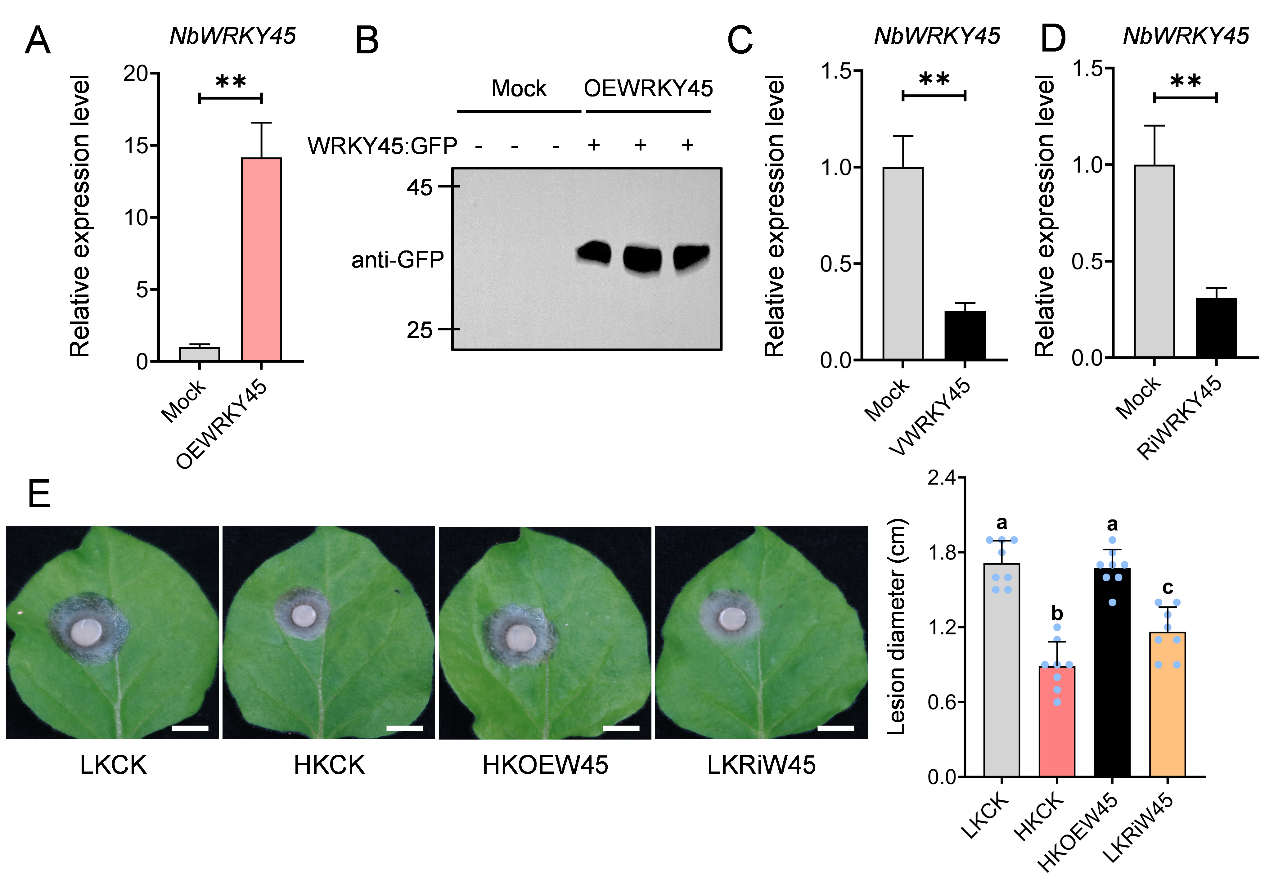


**Figure S8** **Verification and function of stable *NbWRKY45* overexpression or silencing in transgenic *N. benthamiana* seedlings.** **A:** RT-qPCR assay determines the *NbWRKY45* overexpression efficiency in *N. benthamiana*; n = 3. **B:** Westen blot assay confirms the overexpression of NbWRKY45 protein in representative transgenic *N. benthamiana* seedlings. **C:** RT-qPCR confirms the efficiency of VIGs-induced *NbWRKY45* silencing in *N. benthamiana* seedlings; n = 3. **D:** RT-qPCR confirms the efficiency of RNAi-mediated stable *NbWRKY45* silencing in transgenic *N. benthamiana* seedlings. **E:** NbWRKY45 negatively regulates HK-associated resistance in *N. benthamiana* to *B*. *cinerea*. *NbWRKY45* silencing results in increases in resistance in LK *N. benthamiana*, while *NbWRKY45* overexpression decreases resistance in *N. benthamiana* to *B*. *cinerea* even under HK status. Data in **Fig. S8** are presented as the mean ± SD; Different letters and “**” represent significant differences based on *P* < 0.05; Statistical analysis in **Fig. S8A-D** is determined by Student’s *t*-test, and one-way ANOVA followed by post-hoc Tukey test in **Fig. S8E**.


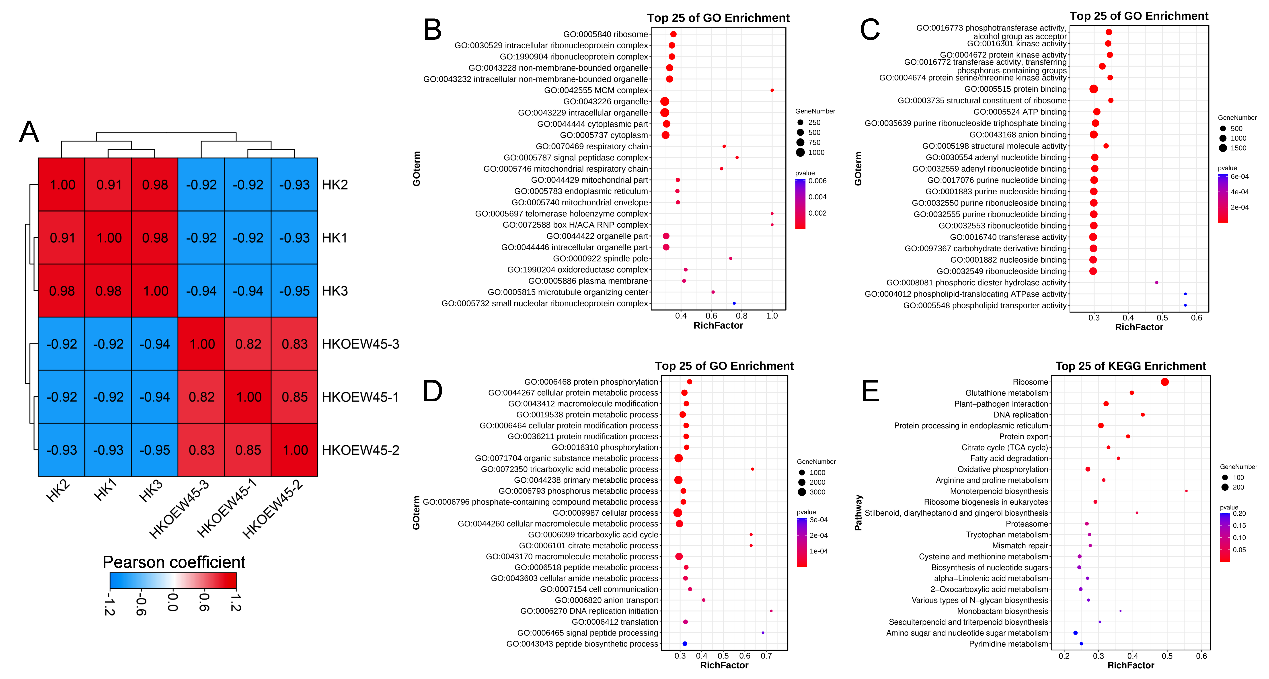


**Figure S9 Transcription landscape in HKMock and HK *N. benthamiana* overexpressing *NbWRKY45*. A:** Correlation analysis for all transcriptome profiles of HKMock and HK *N. benthamiana* overexpressing NbWRKY45 (HKOEWRKY45) based on Pearson algorithm. The high correlation relationship between samples were shown in red. **B-D:** Gene ontology enrichment analysis of DEGs from HKOEWRKY45 vs. HKMock comparison. All DEGs with Gene Ontology matches were assigned to cellular component **(B)**, molecular function **(C)**, and biological process **(D)**. **E:** Pathway analysis of the DEGs from HKOEWRKY45 vs. HKMock comparison. Each plot in the diagram represents a pathway, and the abscissa coordinate and the plot size represent the rich factor and metabolite number of related pathway. The vertical coordinate and plot color represent the P-value (−ln(P-value)) and impact factor of pathway enrichment analysis.

**
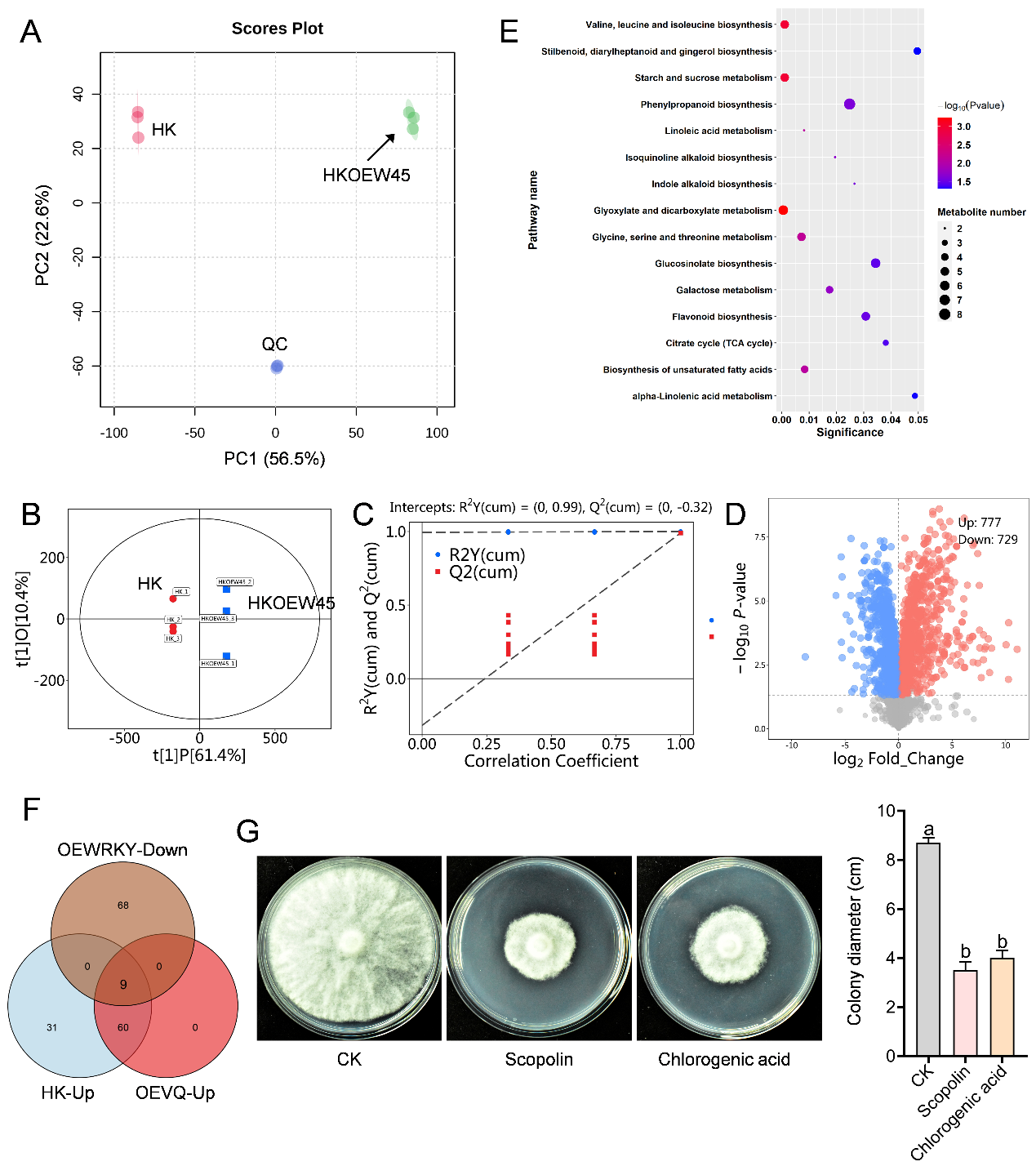
**

**Figure S10 *NbWRKY45* overexpression reshapes the metabolic compositions in HK *N. benthamiana*. A:** PCA scores plot of the samples showing distinct metabolic pattern between HKMock and HK *N. benthamiana* overexpressing NbWRKY45 (HKOEWRKY45). The ellipse represents the 95% confidence interval. The samples from different experimental groups are shown in different color. **B, C:** OPLS-DA scatter plots derived from metabolic profiles of HKMock and HKOEWRKY45 *N. benthamiana*. The validation plots were obtained by using a permutation test that was randomly permuted for 500 times with the first component extracts. **D:** Volcano plot shows the differentially expressed metabolites (DEGs) with log_2.0_fold changed ≥ 1.0 and *P* ≤ 0.05 in HKOEWRKY45 vs. HKMock comparison. Each point in the volcano diagram represents a metabolite, and the horizontal coordinate represents the multiple changes (log_2.0_fold change), and the vertical coordinate represents the *P* value of Student’s t-test (−log_10_P-value). The increased and decreased metabolites are shown in red and blue, respectively. The metabolites labeled by the gray plot are not significant in the comparison. **E:** Pathway enrichment analysis of the downregulated metabolites from HKOEWRKY45 vs. HKMock comparison. Each plot in the diagram represents a metabolic pathway, and the abscissa coordinate and the plot size represent the significance and metabolite number of related pathway. The vertical coordinate and plot color represent the P-value (−ln(P-value)) of enrichment analysis. **F:** Venn diagram identifying the candidate HK- and NbVQ1-associated defensive metabolites that suppressed by *NbWRKY45* overexpression. **G:** Antifungal activity assay of representative phenylpropanoids *in vitro*. Twenty μg/g of scopoline and chlorogenic acid effectively inhibited *P. parasitica* mycelial growth. Colony diameter is presented as ± SD (n = 3; Different letters represent significant differences at *P* < 0.05 based on one‐way analysis of variance followed by post hoc Tukey test).


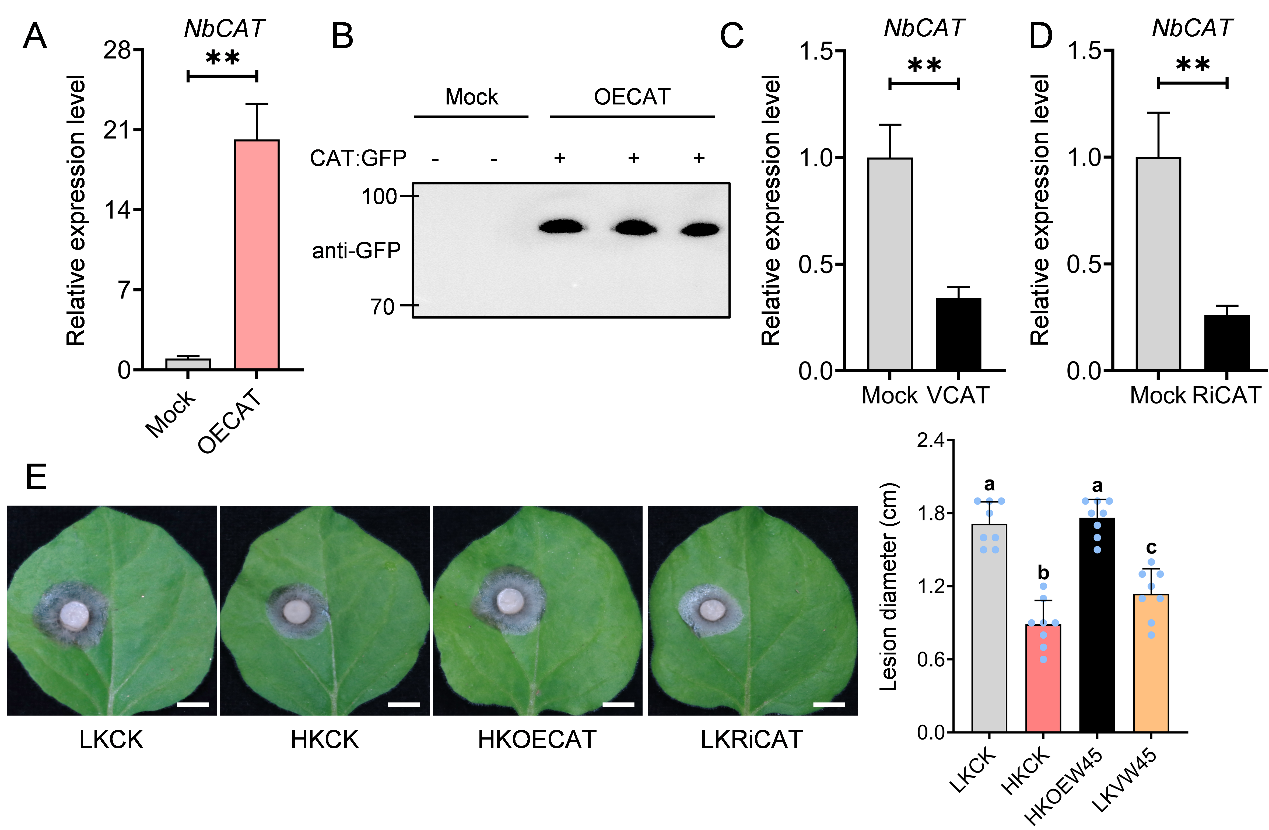


**Figure S11** **Verification and function of stable *NbCAT2* overexpression or silencing in transgenic *N. benthamiana* seedlings.** **A:** RT-qPCR assay determines the *NbCAT2* overexpression efficiency in *N. benthamiana*; n = 3. **B:** Westen blot assay confirms the overexpression of NbCAT2 protein in representative transgenic *N. benthamiana* seedlings. **C:** RT-qPCR confirms the efficiency of VIGs-induced *NbCAT2* silencing in *N. benthamiana* seedlings; n = 3. **D:** RT-qPCR confirms the efficiency of RNAi-mediated stable *NbCAT2* silencing in transgenic *N. benthamiana* seedlings. **E:** *NbCAT2* upregulation is responsible for conferring susceptibility in LK *N. benthamiana* to *B*. *cinerea*. *NbCAT2* silencing results in increases in resistance in LK *N. benthamiana*, while *NbCAT2* overexpression decreases resistance in *N. benthamiana* to *B*. *cinerea* even under HK status. Data in **Fig. S11** are presented as the mean ± SD; Different letters and “**” represent significant differences based on *P* < 0.05; Statistical analysis in **Fig. S11A-D** is determined by Student’s *t*-test, and one-way ANOVA followed by post-hoc Tukey test in **Fig. S11E**.


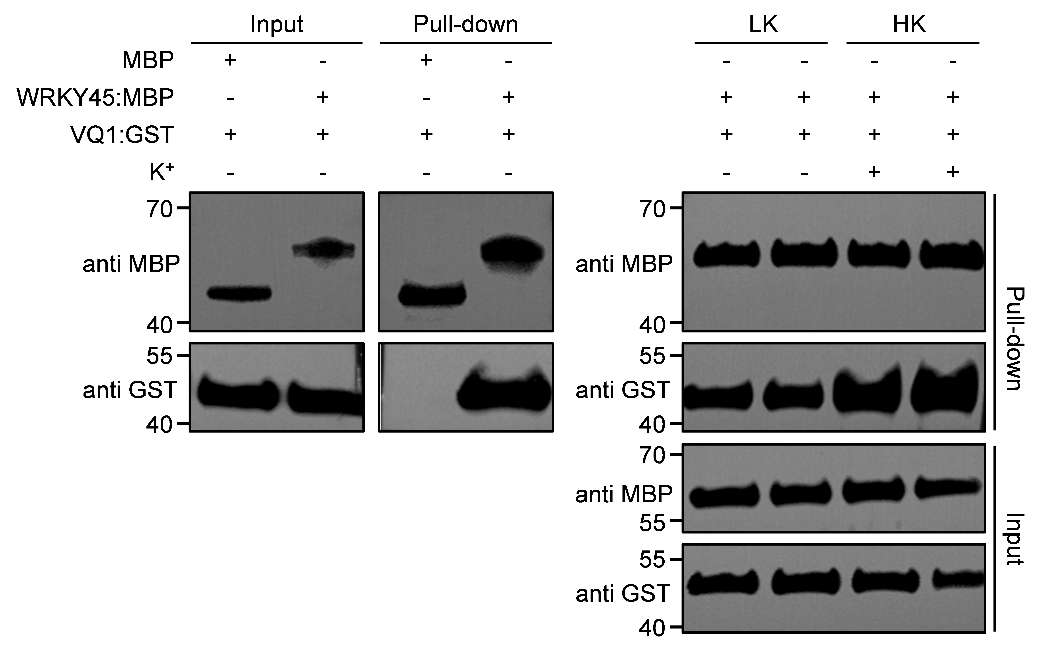


**Figure S12 Pull-down assay determining the interaction of NbWRKY45 and NbVQ1 under HK and LK conditions.** The results showed that adding K^+^ promoted interaction between NbWRKY45 and NbVQ1 *in vitro*. The K^+^ in Pull-down assay was added according to its physiological content in plants. HK: 200mM; LK: 50mM.


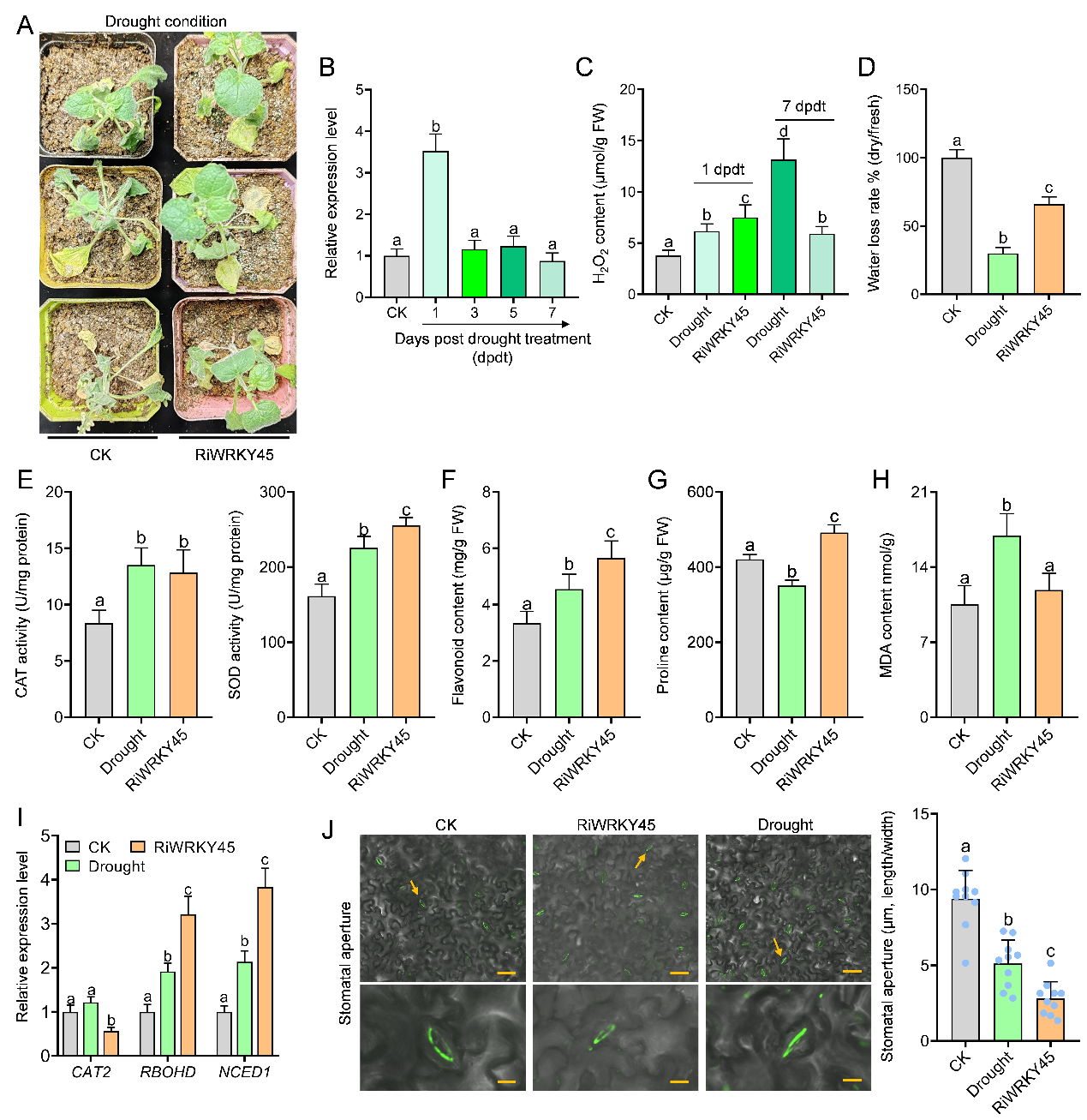


**Figure S13 Silencing of *NbWRKY45* enhanced drought tolerance of *N. benthamiana*. A:** Performance of *N. benthamiana* seedlings with *NbWRKY45* silencing after 10 d of drought treatment. Silencing of *NbWRKY45* enhances drought tolerance of *N. benthamiana* seedlings. **B:** RT-qPCR detecting the expression levels of *NbWRKY45* during treatment of drought stress; dpdt: Days post drought treatment; n = 3. **C:** ROS (H_2_O_2_) contents in *N. benthamiana* seedlings with *NbWRKY45* silencing during treatment of drought stress; n = 3. **D:** The assessment of drought tolerance indicated by water content in *N. benthamiana* seedlings with *NbWRKY45* silencing under drought stress (7 dpdt); n = 3. **E:** CAT and SOD activity in *N. benthamiana* seedlings with *NbWRKY45* silencing under drought stress (7 dpdt); n = 3. **F-H:** The assessment of drought tolerance indicated by flavonoid content (**F**), proline content (**G**) and malondialdehyde (MDA, **H**) content in *N. benthamiana* seedlings with *NbWRKY45* silencing under drought stress (7 dpdt); n = 3. **I:** RT-qPCR analysis detects the expression of ROS-related genes represented by *NbCAT2*, *NbRBOHD* and *NbNCED1* in *N. benthamiana* seedlings with *NbWRKY45* silencing under drought stress (1 dpdt); n = 3. **J:** The observation of stomatal aperture in *N. benthamiana* seedlings with *NbWRKY45* silencing in response to drought stress (1 dpdt); The ROS (H_2_O_2_) in *N. benthamiana* was stained using DCFH-DA probe; Bar = 5 μm; n = 10. Data in **Fig. S13** are presented as the mean ± SD; Different letters in **Fig. S13** represent significant differences at p < 0.05 based on one‐way analysis of variance followed by post hoc Tukey test.


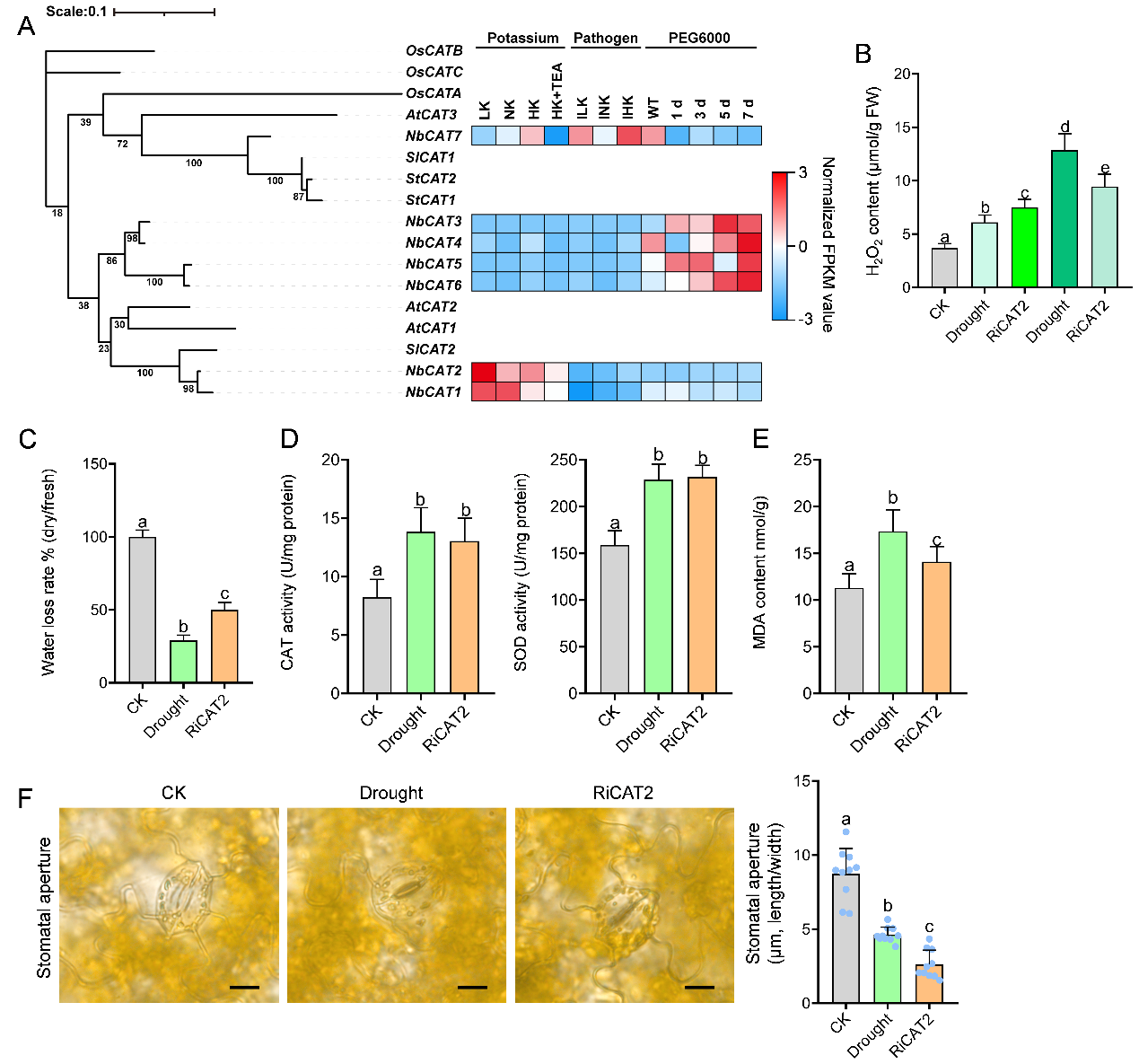


**Figure S14 Function analysis of *NbCAT2* in mediating responses of *N. benthamiana* to drought stress. A:** The phylogenetic analysis of CAT obtained from *N. benthamiana*, *Oryza sativa*, *Solanum lycopersicum*, *Solanum tuberosum* and *A*. *thaliana*. Phylogenetic tree is constructed using RAxML algorithm based on bootstraps = 1000. The Heatmap displaying the expression levels of *NbCATs* in *N. benthamiana* under different conditions. PEG6000 was used to induced drought stress to *N. benthamiana*. **B:** ROS (H_2_O_2_) contents in *N. benthamiana* seedlings with *NbCAT2* silencing under drought stress; n = 3. **C:** Water content in *N. benthamiana* seedlings with *NbCAT2* silencing under drought stress; n = 3. **D:** CAT and SOD activity in *N. benthamiana* seedlings with *NbCAT2* silencing under drought stress; n = 3. **E:** The assessment of drought tolerance indicated by malondialdehyde (MDA) content in *N. benthamiana* seedlings with *NbCAT2* silencing under drought stress; n = 3. **F:** The observation of stomatal aperture in *N. benthamiana* seedlings with *NbCAT2* silencing in response to drought stress; The ROS (H_2_O_2_) in *N. benthamiana* was stained using DCFH-DA probe; Bar = 5 μm; n = 10. Data in **Fig. S14** are presented as the mean ± SD; Different letters in **Fig. S14** represent significant differences at p < 0.05 based on one‐way analysis of variance followed by post hoc Tukey test.


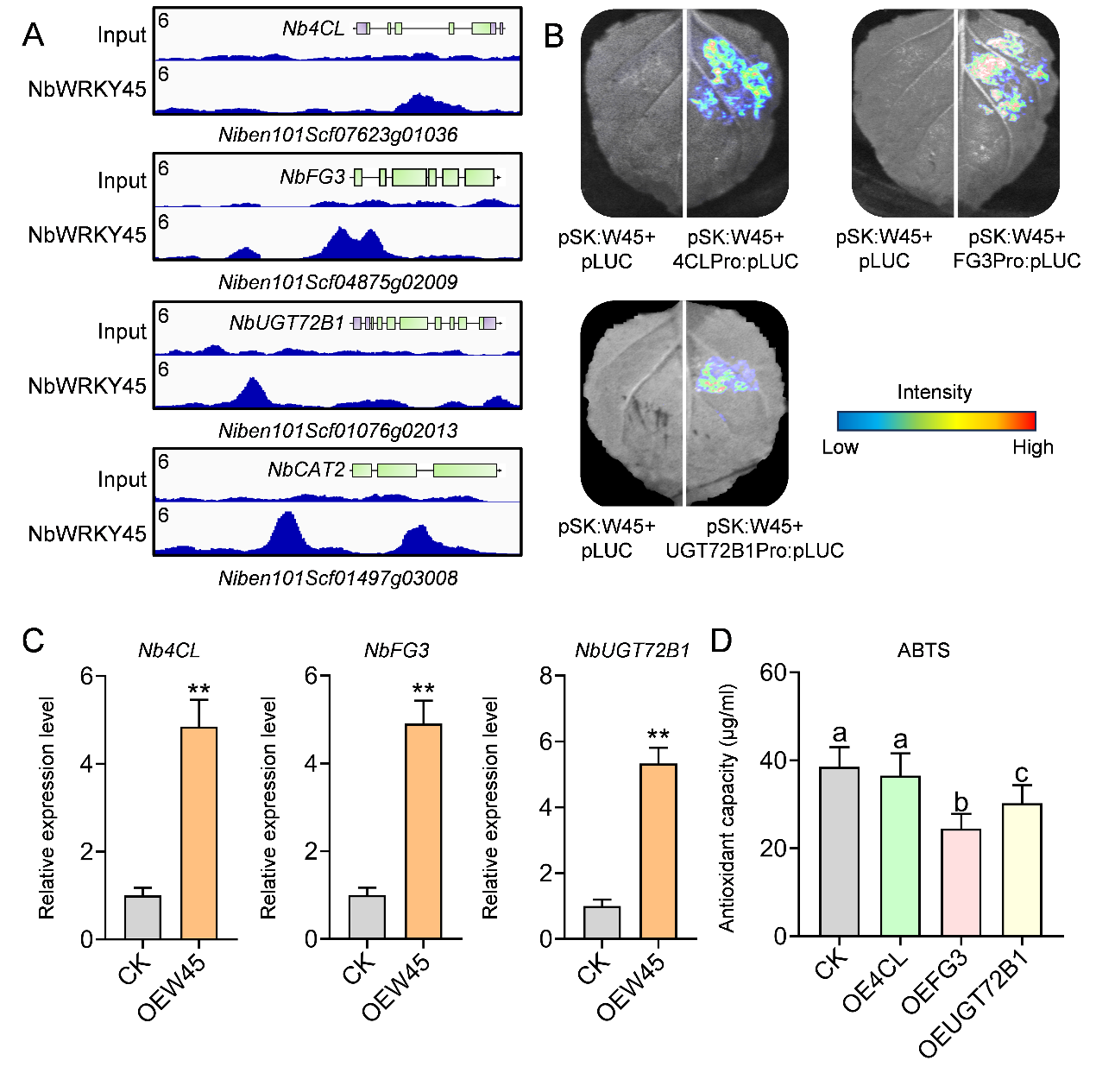


**Figure S15 NbWRKY45 reprograms flavonoid biosynthesis to affect antioxidant capacity in *N. benthamiana*. A:** ChIP-seq analysis revealed NbWRKY45 binding around the promoters of *NbCAT2*, and genes involved in flavonoid synthesis. The gene body is illustrated in each panel. **B:** LUC reporter assay displaying the binding activities of *NbWRKY45* on *Nb4CL*, *NbFG3* and *NbUGT72B1* promoters in *N. benthamiana*. **C:** RT-qPCR detecting the expression levels of *Nb4CL*, *NbFG3* and *NbUGT72B1* in *N. benthamiana* overexpressing *NbWRKY45*; n = 3. **D:** Function of *Nb4CL*, *NbFG3* or *NbUGT72B1* overexpression in affecting antioxidant activities (ABTS scavenging abilities) of *N. benthamiana*. Data in **Fig. S15** are presented as the mean ± SD; Different letters in **Fig. S15** represent significant differences at p < 0.05 based on one‐way analysis of variance followed by post hoc Tukey test.


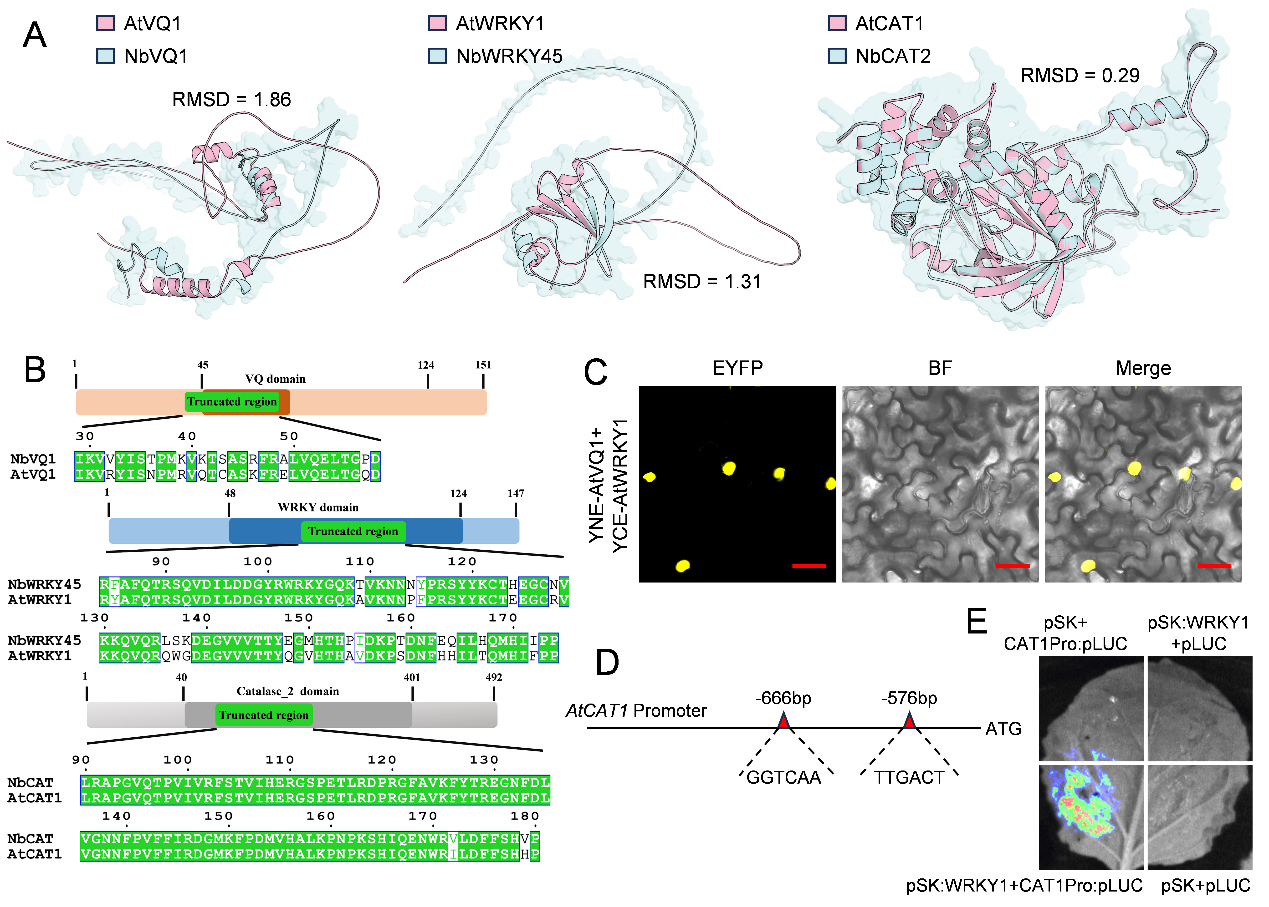


**Figure S16 AtVQ1-AtWRKY1 is conserved in mediating *AtCAT1* expression in *A. thaliana*. A:** Structural comparisons exhibiting high similarity among NbVQ1, NbWRKY45, NbCAT2, and their *A. thaliana* homologs. The proteins from *N. benthamiana* and *A. thaliana* are shown in blue and pink, respectively. The protein structures were predicted using homology model method of Swiss-model online database. **B:** Alignment analysis on amino acid sequence of NbVQ1, NbWRKY45 and NbCAT2 with their homologs from *A. thaliana*. **C:** BiFC assay displays the interaction between AtVQ1 and AtWRKY1 in nucleus; Bar = 20 μm. **D:** Identification of *cis*-elements for WRKY transcription factor in *AtCAT1* promoter. **E:** LUC reporter assay displays the binding affinity of AtWRKY1 on *AtCAT1* promoter.

**Supplemental Tables:**

**Supplemental Table S1** The contents of N, P and K nutrient elements in all *N. benthamiana* seedlings.

**Supplemental Table S2** KEGG pathway enrichment results about gene correlated with NbVQ1, NbVQ7 and NbVQ17 in *N. benthamiana*.

**Supplemental Table S3** Gene ontology enrichment analysis on DEGs from LKOEVQ vs. LK comparison.

**Supplemental Table S4** KEGG pathway enrichment analysis on upregulated DEGs from LKOEVQ vs. LK comparison.

**Supplemental Table S5** Origin data matrix of metabolome profiles from LK and LKOEVQ *N. benthamiana*.

**Supplemental Table S6** KEGG pathway enrichment analysis on upregulated metabolites from LKOEVQ vs. LK comparison.

**Supplemental Table S7** Gene ontology enrichment analysis on DEGs from HKOEWRKY45 vs. HK comparison.

**Supplemental Table S8** KEGG pathway enrichment analysis on downregulated DEGs from HKOEWRKY45 vs. HK comparison.

**Supplemental Table S9** Origin data matrix of metabolome profiles from HKOEWRKY45 and HK *N. benthamiana*.

**Supplemental Table S10** KEGG pathway enrichment analysis on downregulated metabolites from HKOEWRKY45 vs. HK comparison.

**Supplemental Table S11** Predication results of cis-elements in *NbCAT2* promoters.

**Supplemental Table S12** Predication results of cis-elements in *NbWRKY45* promoters using PlantCARE database.

**Supplemental Table S13** Predication results of cis-elements in *AtCAT1* promoter.

**Supplemental Table S14** Sequence information of all primers and probes.

**Supplemental Table S15** FoldX predication of the interaction affinity between NbVQ1, NbWRKY45 and NbCAT2 promoter.

**Supplemental Table S16** Interaction targets of NbVQ1 from IP-MS results.
